# Quantifying *Leishmania* Metacyclic Promastigotes from Individual Sandfly Bites Reveals the Efficiency of Vector Transmission

**DOI:** 10.1038/s42003-019-0323-8

**Published:** 2019-02-28

**Authors:** Emilie Giraud, Oihane Martin, Laith Yakob, Matthew Rogers

**Affiliations:** 1 Department of Immunology and Infection, Faculty of Infectious Tropical Diseases, London School of Hygiene and Tropical Medicine, Keppel Street, London, WC1E 7HT UK; 2 Department of Disease Control, Faculty of Infectious Tropical Diseases, London School of Hygiene and Tropical Medicine, Keppel Street, London, WC1E 7HT UK; 3 Present Address: Institut Pasteur, 25-28 rue du Dr Roux 75015, Paris, France

## Abstract

Predicting how *Leishmania* will respond to control efforts requires an understanding of their transmission strategy. Using real-time quantitative PCR to quantify infectious metacyclic and non-metacyclic forms in mouse skin from single sandfly bites we show that most transmissions were highly enriched for infectious parasites. However, a quarter of sandflies were capable of transmitting high doses containing more non-infectious promastigotes from the vector’s midgut. Mouse infections replicating “high” to “low” quality, low-dose transmissions confirmed clear differences in the pathology of the infection and their onward transmissibility back to sandflies. Borrowing methods originally developed to account for exposure heterogeneity among hosts, we show how these high-dose, low-quality transmitters act as super-spreading vectors, capable of inflating *Leishmania* transmission potential by as much as six-fold. These results highlight the hidden potential of transmission of mixed *Leishmania* promastigote stages on disease prevalence and the role of dose heterogeneity as an underlying strategy for efficient transmission.

## Abstract (web summary)

Emilie, Giraud et al. show that high-dose, low-quality transmitters act as super-spreading parasite vectors, inflate *Leishmania* transmission potential by six-fold. This study highlights the role of *Leishmania* promastigote dose heterogeneity as an underlying strategy for efficient parasite transmission.

## Introduction

Leishmaniasis is a parasitic protozoal disease caused by the bite of an infected phlebotomine sandfly. It afflicts 12 million people in 98 countries and is responsible for 30,000–40,000 deaths with 1.2 million new infections annually^1^. A lack of a vaccine combined with a limited choice of drugs, which have toxicity issues and a growing incidence of drug resistance, place vector control as an important part of the future global elimination strategy^2^. Underpinning this is the need to understand the biology of *Leishmania* transmission and to model the transmission strategies of these parasites in both the lab and field^2–4^. Despite this need, we have poor information on the natural heterogeneity of *Leishmania* transmission, and currently no tools to measure transmission intensity in the field.

In the midgut of the sandfly, *Leishmania* develop into infective metacyclic promastigotes, a process termed metacyclogenesis. To achieve this, *Leishmania* must transform through various non-infectious stages of promastigotes (procyclic, nectomonad, leptomonad and haptomonad promastigotes). Collectively, their role is to replicate and colonize the sandfly forming a biological plug of parasites, which block the anterior midgut and modify the feeding behavior of the vector^5–8^. To do this they secrete filamentous proteophosphoglycan (fPPG), which condenses into the promastigote secretory gel (PSG), forcing the sandfly to regurgitate parasites during bloodfeeding^9–11^. One aspect of *Leishmania* transmission that has received very little attention is the composition of the parasite dose – the proportion of metacyclics delivered by bite. For leishmaniasis, this is a key question since parasites are likely to originate, via regurgitation, from the midgut where both metacyclics and non-metacyclics are embedded in PSG^11^. Currently, it is assumed that all infected sandflies transmit near-homogenous populations of metacyclics, based on a small number of studies which relied on determining the morphology of parasites recovered from capillary-feeding, membrane-feeding or from exudates squeezed from fresh bites^9,12–18^. Although informative, they do not fully replicate the natural feeding processes of the fly or the dynamic of deposition of the parasites into living skin.

Here we developed a real-time quantitative PCR (RTqPCR) strategy to quantify the number of metacyclic and non-metacyclic promastigotes within and delivered by individual sandflies to living mice. Contrary to current models, we show that there is heterogeneity in both the number and proportion of metacyclics transmitted by bite. Our study also shows that for *Leishmania mexicana* this infectiousness changes as the parasites mature in the vector *Lutzomyia longipalpis*, and can be affected by its feeding history and intrinsic factors during metacyclogenesis, such as the accumulation of the PSG plug. Strikingly, changes to the composition of the infectious dose from sandflies was shown to dramatically impact on the pathogenesis of cutaneous leishmaniasis and its infectiousness towards other sandflies. Mathematical modelling predicts that heterogeneity in dose composition may increase *Leishmania* transmission rate six-fold, highlighting that a proportion of sandflies act as super-spreading vectors.

## Results

### RTqPCR to quantify metacyclics in sandflies and skin

**Fig. 1:**
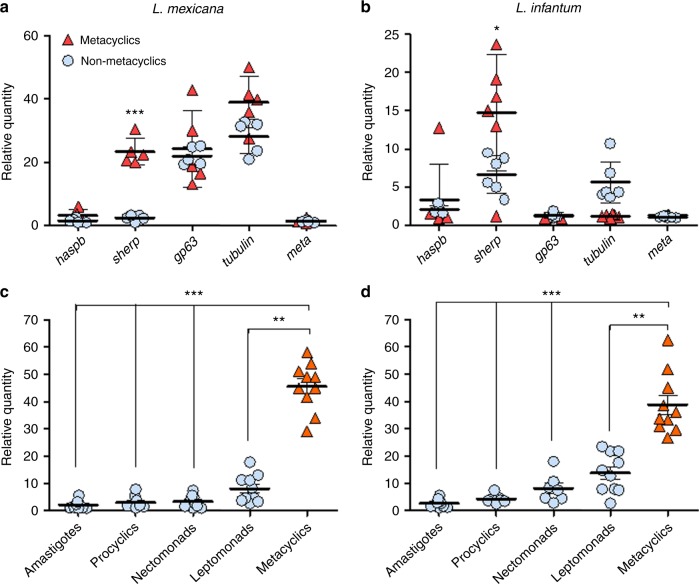
Quantifying *L. mexicana* and *L. infantum* metacyclic promastigotes using *sherp* expression. **a**, **b** In vitro cultured *L. mexicana* and *L. infantum* non-infectious nectomonad promastigotes and infectious metacyclic promastigotes were assessed for their expression of a panel of metacyclic-associated transcripts using RTqPCR. Data shown are representative of 3 independent experiments. **c**, **d** Sandfly-derived *L. mexicana* and *L. infantum* parasites, enriched for non-infectious procyclic promastigotes (20 h p.i. *L. mexicana*, 40 h p.i. *L. infantum*); nectomonad promastigotes (40 h p.i. *L. mexicana*, 72 h p.i. *L. infantum*); leptomonad promastigotes (120 h p.i. *L. mexicana*, 168 h p.i. *L. infantum*) and metacyclic promastigotes (196 h p.i. *L. mexicana*, 288 h p.i. *L. infantum*) were assessed for their expression of *sherp*. Data shown are pooled from 4 independent experiments showing the expression from 7 × 10^5^–1 × 10^6^ cells for each point. Solid lines represent means ± 1 s.e.m. Asterisks indicate values that are statistically significant (*P ≤ 0.05, **P ≤ 0.005, ***P ≤ 0.0005) using a two-sided unpaired t-test

*Lutzomyia (Lu.) longipalpis* sandflies were infected with *Leishmania (L.) mexicana* or *Leishmania infantum*, agents of zoonotic cutaneous and visceral leishmaniasis, and maintained until the infections had matured and undergone metacyclogenesis^19,20^. Individual flies were allowed to feed on the ears of BALB/c mice once and the bite site was analyzed by RTqPCR for the number of *Leishmania* parasites and the proportion of metacyclic promastigotes. Initially, a number of previously identified metacyclic-enriched *Leishmania* transcripts (Supplementary Table 1) were screened against, culture-derived non-infectious, *L. mexicana* and *L. infantum* nectomonads and infectious metacyclics (Fig. 1a, b). Following this, titration of *L. mexicana* and *L. infantum* metacyclics confirmed transcripts for small hydrophilic endoplasmic reticulum-associated protein (*sherp*)^21–23^ displayed sufficient specificity and sensitivity to discriminate and quantify the infective forms of these two species of *Leishmania*. In addition, metacyclics and nectomonads expressed similar amounts of small subunit ribosomal RNA (*ssrRNA*) transcripts (Supplementary Figure 1), confirming its ability to quantify infectious and non-infectious forms alike. As a negative control, neither of the promastigote forms expressed the amastigote-specific transcript, *amastin* (Supplementary Figure 1). By combining these parasites with a biopsy of mouse ear skin or an uninfected sandfly midgut we determined the abundance of *ssrRNA* and *sherp* transcripts and they formed our calibration curves for parasite and metacyclic quantification throughout the rest of the study. To determine the threshold sensitivity of detection of this method, we established a standard curve using serial 10-fold dilutions of *L. mexicana* or *L. infantum* parasites ranging from 10^6^ to 1 parasite per reaction (Supplementary Figure 2 and Supplementary Table 2). Using *ssrRNA*, we were able to reliably detect down to 1 *Leishmania* cell per reaction for both species. Mean standard curves were calculated from five independent experiments in triplicate and was linear over the 7 log-dilutions of *L. mexicana* and *L. infantum* parasites with a correlation coefficient of 0.9927 and 0.9868, respectively. The *ssrRNA* and *sherp* RTqPCR proved highly reproducible over the entire range of parasite numbers and proportions of metacyclics, showing intra- and inter-assay coefficients of variation lower than 1.22% and 11.3%, respectively, and sensitivity to 1.4 pg *Leishmania* cDNA in as much as 2 μg mouse ear or 100 pg sandfly midgut cDNA (Supplementary Figures 2, 3, 4, 5 and Supplementary Tables 2, 3, 4, 5). Importantly, *ssrRNA* expression was very similar over the range of metacyclic proportions for all parasite numbers tested, confirming equal expression in non-infectious and infectious promastigotes (Supplementary Figure 3 and Supplementary Table 3).

To validate this method in sandflies we performed the *sherp* RTqPCR on sequential days of *L. mexicana* and *L. infantum* infection in *Lu. longipalpis*, and compared this to direct counting of promastigotes from midgut homogenates and determining the proportion of metacyclics from Giemsa-stained smears (Supplementary Figure 6a–d). The *sherp* RTqPCR showed close agreement with the established morphological method of quantifying metacyclics as the midgut infections matured. Going further, we tested the ability of the *sherp* RTqPCR to discriminate the non-infectious promastigote forms of *L. mexicana* and *L. infantum* from metacyclics obtained from infected sandflies. Amastigotes from infected mouse skin or spleens were used as negative controls (Fig. 1c, d). Collectively, these results show that for these species of *Leishmania* the combination of *ssrRNA* and *sherp* RTqPCR provides a robust method to discriminate and quantify metacyclics and non-metacyclics both in sandflies and in the skin following transmission. However, the use of the qPCR based on *sherp* expression may not be applicable for other *Leishmania* species or strains, and we would advise that prior validation is necessary before use.

### Infected sandflies transmit highly enriched doses of metacyclics

**Fig. 2:**
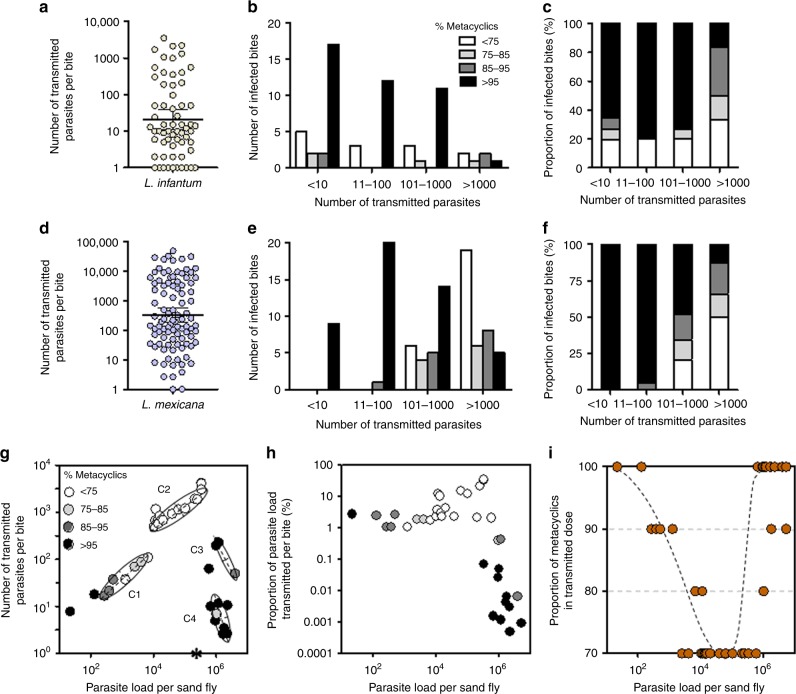
Composition of infective dose from individual sandfly bites. **a**–**i** *Lutzomyia longipalpis* sandflies were infected and allowed to bite the ear of a BALB/c mouse once at 10 days p.i. for *L. infantum* (**a**–**c**) or 8 days p.i. for *L. mexicana* (**d**–**h**). Each bite was assessed for the total number of parasites by *ssrRNA* RTqPCR (**a**, **d**) and the proportion of metacyclics by *sherp* RTqPCR (**b**, **c**, **e**–**i**). Thick solid lines represent geometric means ± 95% C.I. **g** The relationship between the quantity of *L. mexicana* parasites in the prefed midgut of *Lu. longipalpis* and the infectious dose transmitted by each fly. Two-dimensional Gaussian mixture cluster analysis identifies 4 clusters centred on the mean, plotted as an ellipse representing the standard deviation. The asterisk on the x-axis represents the infection of one fly from this subset that didn’t transmit any parasites. **h** The parasite dose as a function of the midgut infection intensity. The relationship between the pre-feeding parasite load of flies in **g** and the proportion of the load transmitted. **g**, **h** The proportion of metacyclics delivered by each fly bites was determined and is presented as different coloured symbols. **i** The quality of parasite dose as a function of midgut infection intensity. The dashed black line represents a loess curve of the data points. Data for (**a**–**f**) are pooled from 4 independent experiments. Results from (**g**–**i**) are from 1 experiment

Studies that have determined the number of *Leishmania* promastigotes delivered by individual sandflies have highlighted the large variability of parasite transmission^19,24,25^. In addition to determining the number of *L. infantum* and *L. mexicana* promastigotes delivered by individual infected *Lu. longipalpis* sandflies (the dose); we used our *sherp* RTqPCR to determine the metacyclic/non-metacyclic composition of each transmission. *ssrRNA* RTqPCR revealed that *L. mexicana*- and *L. infantum*-infected flies could transmit a mean 4111 and 268 promastigotes, respectively (Fig. 2a, d, Supplementary Table 6). Doses of *L. infantum* and *L. mexicana* displayed a distinct bimodal distribution; similar to other studies that have investigated *Leishmania major* transmitted by *Phlebotomus papatasi*^20^ or *L. infantum* transmitted by *Phlebotomus perniciosus* and *Phlebotomus duboscqi*^21^. Confirming these earlier reports, the majority of bites resulted in low doses, with 74% (47/62 bites) of *L. infantum*-infected flies and 58% (46/80 bites) of *L. mexicana*-infected flies delivering less than 10^2^ or 10^3^ promastigotes, respectively. *Sherp* RTqPCR revealed that in general *Leishmania*-infected flies delivered highly enriched doses of metacyclics, with an average of 88% *L. infantum* and 85% *L. mexicana* metacyclics transmitted to skin per bite. However, in these flies it was low-dose transmissions, comprising the majority of *L. infantum* or *L. mexicana* bites, that were the most enriched for infective forms, such that 83% *L. infantum* and 76% *L. mexicana* bites transmitting less than 10^2^ or 10^3^ parasites, respectively, contained an average 89% and 92% metacyclics (Fig. 2b–f). By contrast, high doses of these infections (i.e., > 10^3^ parasites) contained proportionally less metacyclics (“metacyclic-poor”), delivering an average 63% *L. infantum* and 68% *L. mexicana* infective forms per bite.

To gain further insight into the relationship between the sandfly infection and both the quantity and quality of the infectious dose we applied a Gaussian mixture cluster analysis on log-transformed total parasite counts to a separate set of 35 *L. mexicana*-infected flies, which had their midgut loads determined immediately following the infectious bite. A sandfly’s pre-feed infection load was determined by combining the dose of parasites with the parasitaemia in the sandfly midgut^24^. This analysis detected four clusters with normal distribution and equal variance (Fig. 2g, Supplementary Table 7). Two clusters displayed a strong positive correlation between dose and midgut infection intensity and two that displayed a negative correlation. Interestingly, the flies in which the relationship between parasite load and transmitted dose inverted were those with the highest infections (geometric mean 1.41 × 10^6^; compared to the other flies: geometric mean 4.23 × 10^4^). Notably, these flies had atypically high infections but still in line with loads occasionally recorded from field-caught flies^26,27^. When the proportion of metacyclics in each dose was determined, this revealed that the vast majority of flies (91%, 21/23 flies) capable of transmitting low to intermediary doses of < 5 × 10^2^ parasites, representing all flies from cluster 1, 3 and 4, deposited metacyclic-enriched doses of ≥ 75% metacyclics. These flies harbored low to intermediary (cluster 1) or very high (cluster 3 and 4) infection intensities. In contrast, poor-quality transmission ( < 75% metacyclics/bite) was almost an exclusive feature of cluster 2 flies, despite containing high infection levels and transmitting the highest doses (Fig. 2g, Supplementary Table 7).

As *Leishmania* transmission is likely to be by regurgitation, especially from heavily infected flies^9,12,24^, we next calculated the proportion of the original gut infection that was deposited with each bite (Fig. 2h, Supplementary Table 7). We found that the high-dose, poor-quality transmitters from cluster 2 disgorged the highest proportion of their infection (geometric mean 7.7 %). By contrast, those flies able to transmit low to intermediary doses exhibited consistently high-quality transmission and deposited a geometric mean of 2.42% of their infection. However, at the extreme end of the spectrum, flies with unusually high midgut infections of 10^5^–10^6^ parasites (from cluster 3 and 4) transmitted doses containing > 95% metacyclics but could only disgorge a geometric mean 0.008% of their initial load, and deposit low doses of parasites. For these flies, the relationship between sandfly infection intensity and the proportion of metacyclics in the transmitted dose (Fig. 2i) demonstrated that dose quality dropped rapidly when the sandfly infection reached 5.4 × 10^3^ then steeply recovered when the infection reached 3.6 × 10^5^ promastigotes per midgut (at 80% metacyclics per bite). This suggests that dose quality is highest during early metacyclogenesis or later when the infection is very large. In between, flies that harbour moderate infections are more likely to regurgitate higher doses containing proportionally more non-metacyclics.

### Composition of the infectious dose changes during metacyclogenesis

**Fig. 3:**
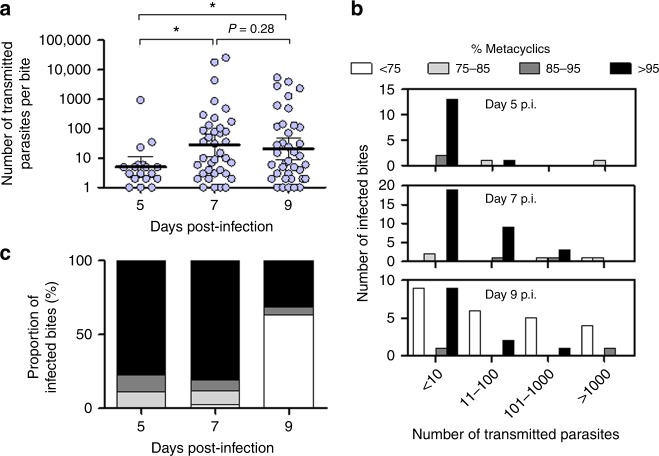
Composition of the infective dose as *Leishmania* develop in sandflies. **a**–**c** *Lutzomyia longipalpis* sandflies were infected with *L. mexicana* amastigotes and on days 5, 7 and 9 p.i. were allowed to bite the ears of BALB/c mice once. **a** The total number of parasites (*ssrRNA*) and (**b**, **c**) the proportion of metacyclics (*sherp*) transmitted to the bite site was determined by RTqPCR. n = 12–16/day. Bars represent the geometric mean ± 95% C.I. Data shown are pooled from 4 independent experiments. Asterisks indicate values that are statistically significant (*P ≤ 0.05) between the indicated groups using a two-sided unpaired t-test

*Leishmania* metacyclogenesis starts after the parasites detach from the sandfly midgut epithelium following bloodmeal defecation. For *L. mexicana* in *Lu. longipalpis* metacyclogenesis typically occurs from day 4 and reaches a plateau by day 8 post-infection (p.i.), achieving 40–60% of the total infection (Supplementary Figure 6a)^8,20^. To see if there was an optimal window of transmission we assessed *L. mexicana* transmission from *Lu. longipalpis* as the infections underwent metacyclogenesis from day 5–9 p.i. (Fig. 3). During this time, the infection level and proportion of metacyclics accumulated in midguts as expected (Supplementary Table 8), indicating that flies with atypically high infections, as experienced in Fig. 2g, h, were absent. In the bite, the numbers of promastigotes delivered increased 10-fold from day 5 to 7, and then dropped slightly from day 7 to 9 p.i. (Fig. 3a). Despite the low numbers of parasites, the majority (14/18 bites, 78%) of day 5 p.i. flies deposited highly metacyclic-enriched doses ( > 95%; Fig. 3b, c), which continued into day 7 p.i. (31/38 bites, 82%) except that the average dose and range of doses increased. As metacyclogenesis proceeded the proportion of flies transmitting less enriched doses (24/38 bites, 63%) became more apparent, such that metacyclic-poor transmissions ( < 75%) were only detected from day 9 p.i. flies. In this cohort, flies capable of transmitting large doses ( ≥ 10^3^ promastigotes) were more prominent, representing an eighth of flies (Fig. 3b).

### Increasing metacyclic purity with multiple successive bites

**Fig. 4:**
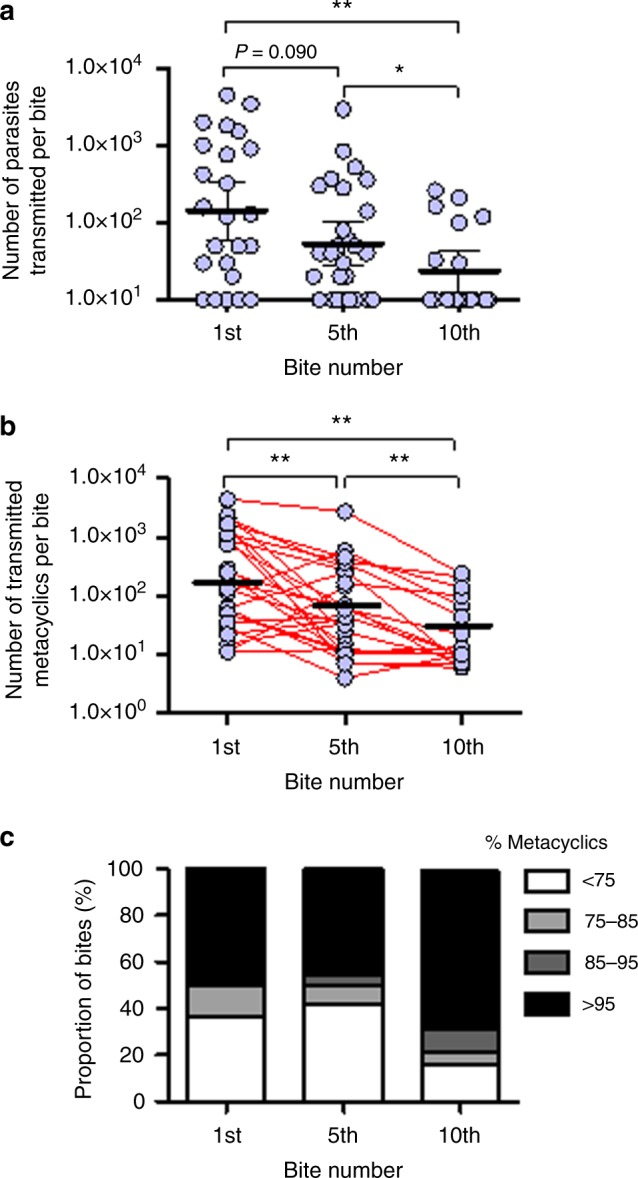
Composition of the infective dose following multiple sandfly bites. **a**–**c** *Lutzomyia longipalpis* sandflies were infected with *L. mexicana* amastigotes and on day 7 or 8 p.i. were allowed to bite the ears of BALB/c mice ten consecutive times. For each fly, the 1st, 5th and 10th bite were assessed for the total number of transmitted parasites (*ssrRNA*, **a**), number of transmitted metacyclics (*sherp*, **b**) by RTqPCR and the relative proportion of metacyclics present in each bite (**c**). Data pooled from 4 independent experiments, *n* = 15–25/group. Bars represent the mean ± s.e.m. Asterisks indicate values that are statistically significant (*P ≤ 0.05, **P ≤ 0.005) using a two-sided Wilcoxon paired t-test

Leishmaniasis tends to be a focal disease, suggesting that there might be an influence of parasite infection on sandfly biting behavior^4,28^. Using the *sherp* RTqPCR we took the opportunity to examine the composition of the dose from multiple infective bites, taken in rapid succession from the same fly to assess the efficiency of transmission, which may occur in infection hot-spots. The quantity and composition of the first, fifth and tenth bites from 53 day 7 or 8 *L. mexicana*-infected *Lu. longipalpis* to the ears of BALB/c mice were investigated. To encourage multiple feeding, each fly was allowed to feed for 45 s, as this was roughly a fifth of the time required for infected *Lu. longipalpis* to take a full bloodmeal^8^. Figure 4 shows that there was 68% drop in the mean number of transmitted promastigotes (Fig. 4a) and metacyclics (Fig. 4b) between the first and fifth bites. Although the range of doses delivered by these flies was somewhat lower than the previous experiments (Fig. 2), this probably reflects the fact that these flies were allowed to feed for less time. By the tenth bite we observed a significant drop of 92% in the mean number of transmitted parasites (*P* < 0.05), and metacyclics compared to the first bite (Fig. 4a, b). However, proportionally, the number of bites containing highly enriched doses of metacyclics ( > 95%) rose sharply from 48% to 75%, between the 5^th^ and 10^th^ bite (Fig. 4c). These results demonstrate that infected sandflies remain infectious for many, consecutive, bites (up to ten and possibly beyond) and the quality of transmission may improve with each bite. This, combined with an increase in a sandfly’s feeding persistence during metacyclogenesis^20^, may promote the high efficiency of *Leishmania* transmission even if the population of infectious flies is low.

### PSG blockage promotes more metacyclic deposition with multiple bites

**Fig. 5:**
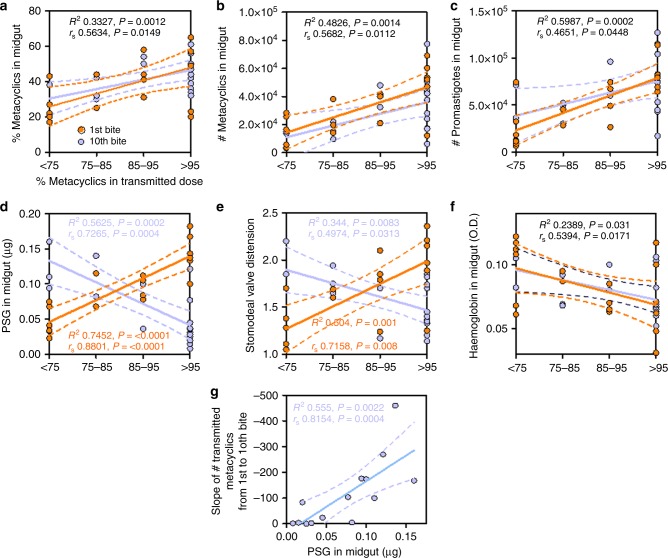
Correlates of sandfly infection with dose composition. **a**–**g** Following the 1st or 10th infectious bite, *L. mexicana*-infected *Lu. longipalpis* sandflies were dissected and their parasite infections assessed. For each fly the proportion of metacyclics in the infectious dose is plotted against: (**a**) the percentage and (**b**) total number of metacyclics in the midgut of prefeed flies, (**c**) total midgut parasite burden in prefeed infected flies, (**d**) the relative quantity of PSG in the midgut remaining after the feed, (**e**) distension of the stomodeal valve (compared to age-matched, bloodfed, uninfected flies) and **f** the quantity of haemoglobin imbibed. Results in orange are from flies allowed to bite once and blue for flies allowed to bite 10 times. **g** Transmitted dose as a function of the PSG blockage in the midgut. The slope of the number of metacyclics transmitted from the 1st to the 10th bite of flies allowed to bite 10 times is plotted against the relative quantity of PSG remaining in the midgut after the last bite. Data pooled from 4 independent experiments. Solid lines represent linear regression line of best fit and dotted lines represent the 95% confidence interval. Correlation coefficients were generated by Spearman-rank correlation and are colour-coded. Those in black represent correlation coefficients from the combination of single- and 10-bite flies due to their similarity

Flies from the multiple bite experiments in Fig. 4 and another set of flies allowed to bite only once were dissected and processed to determine a number of parameters pertinent to infection and plotted against the proportion of transmitted metacyclics^5,8,13,20,24,29^ (Fig. 5).

Corroborating the work of Stamper and colleagues^29^ on *L. major* transmission from *P. duboscqi*, meta-analysis of *L. mexicana* infectious bites from *Lu. longipalpis* revealed that, overall, the proportion and total number of metacyclics per fly correlated with the proportion of metacyclics from flies transmitting their 1st and 10th infectious bite (Fig. 5a, b). The same association was also seen with the total parasite midgut burden (Fig. 5c). However, flies transmitting > 95% metacyclics displayed a greater spread of values, indicating that a proportion of flies with small or immature infections could also deliver high-quality doses, although the majority came from flies with large infections and higher proportion of metacyclics in their midguts. The sandfly infection parameter most closely associated with the proportion of metacyclics in the transmitted dose was the amount of PSG present in the midgut of flies delivering their 1^st^ or 10^th^ infectious bite (Fig. 5d). Interestingly, the multiple bite flies yielded a negative correlation between post-feed midgut PSG levels and the quality of the 10th bite, whereas bites from flies exposed only once to mice showed a strong positive correlation. Stomodeal valve distortion, linked to the accumulation of PSG and blockage of the anterior midgut returned a similar result (Fig. 5e), whilst the amount of blood obtained during the transmissions displayed a negative correlation for flies taking multiple bites and those fed once (Fig. 5f). Wing area, a commonly used proxy for (pre-bloodfed) dipteran body size^30^, did not return any correlation with the proportion of egested metacyclics, confirming that this was not a confounding factor in our study (Supplementary Figure 7).

Meta-analysis of the loss of metacyclics from sequential infectious bites, by comparing the slope between the first and last (10^th^) transmission, revealed that the amount of PSG blocking the sandfly midgut correlated with deposition of metacyclic forms (Fig. 5g). Collectively, these results indicate that, in addition to the proportion of parasites that have differentiated to the infectious metacyclic form, formation of the PSG blockage is an important component of vectorial competency and predictor of efficient transmission.

### Dose composition influences leishmaniasis and onward transmission

To test the consequence of dose quality for infection of the mammalian host we mimicked high- to low-quality *L. mexicana* transmissions using 5 × 10^2^ sandfly-derived metacyclics inoculated into the ear dermis of BALB/c mice on their own (high-quality, 100% metacyclic dose), or premixed with 2.5 × 10^2^ or 5 × 10^2^ sandfly-derived nectomonads (low-quality, 75% and 50% metacyclic doses). These infections used 5 × 10^2^ promastigotes as this is close to the geometric mean dose of *L. mexicana* parasites delivered by *Lu. longipalpis* bite 7–8 days p.i. (Fig. 2a). In addition, a fourth group of mice received a total of 1 × 10^3^ nectomonads and a fifth group 1 × 10^3^ metacyclics only.

**Fig. 6:**
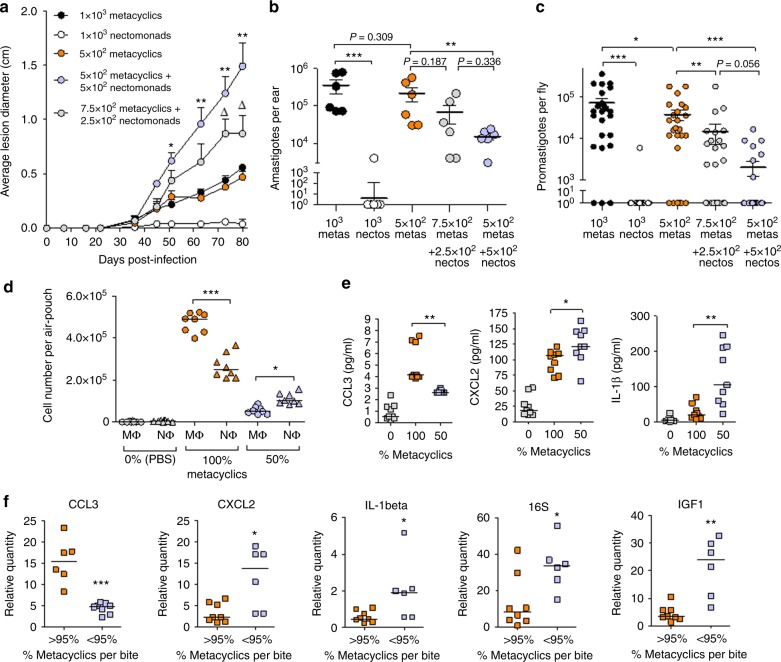
Composition of *Leishmania* dose on infectivity and transmission potential. **a** Influence of proportion of *L. mexicana* metacyclic promastigotes on the course of cutaneous infection in BALB/c mice. Low quality and high quality doses of *L. mexicana* were generated using sandfly-derived metacyclic promastigotes mixed with sandfly-derived, nectomonad promastigotes (75% and 50% metacyclic promastigotes) or saline (100% metacyclic promastigotes). Promastigotes were injected i.d. into the ears of 10 BALB/c mice and the course of infection monitored weekly by measuring the diameter of any lesion. **b** Final parasite burden of infections from **a** harvested at 80 days p.i. **c** Day 4 p.i. promastigote burdens of sandfly midguts (n = 25–30) from flies allowed to take a full bloodmeal from ear lesions in **a** at the end of the infection. **d** Macrophage (MΦ) and Neutrophil (NΦ) cell recruitment profiles to sterile air-pouches on BALB/c mice injected with PBS, 1 × 10^3^ sand fly-derived metacyclics alone (100%) or an equal mix with 5 × 10^2^ sandfly-derived nectomonads (50%) 48 h p.i. **e** Production of chemokines CCL3, CXCL2 and pro-inflammatory cytokine IL-1β from air-pouch supernatants in **d** determined by Luminex ELISA. **f** Expression of chemokines *CCL3* and *CXCL2*; cytokines *IL-1β* and *IGF1* and detection of bacteria via 16S *rRNA* in BALB/c ears receiving infected sandfly bites of 1,000 parasites or more up to 1 h post-bite; determined by RTqPCR. The data represents a single experiment following 3 independent similar iterations. Bars represent means ± s.e.m. Asterisks and delta symbols indicate values that are statistically significant from infection with 1 × 10^3^ metacyclics alone (**a**) or between groups, as indicated (Δ*P* ≤ 0.05, *P *≤* 0.05, **P ≤ 0.005, ***P ≤ 0.0005) using a two-sided unpaired *t* test

Infections from doses of 100% nectomonads confirmed that this stage of *Leishmania* is poorly infectious to mice compared to metacyclics (Fig. 6a, b). However, strikingly, infections incorporating nectomonads in the infectious inoculum (mimicking low-quality doses) caused exacerbated cutaneous pathology. Lesions evolved with faster kinetics compared to those infections from high-quality doses (Fig. 6a), resulting in much larger lesions. The final amastigote burdens revealed the advantage of a high-quality dose to the overall parasite infection in mice (Fig. 6b) as lesions resulting from 5 × 10^2^, 100% metacyclics harbored an average of 3- or 15-fold more parasites compared to those initiated with 75% and 50% mix of metacyclics and non-infectious nectomonads, respectively (average amastigote burden: 2.2 × 10^5^ vs. 6.8 × 10^4^ (75%) 1.5 × 10^4^ (50%)). To assess the transmission potential of these groups of lesions back to sandflies we allowed uninfected flies to feed on them immediately before the lesions were harvested. To reduce variability between the two groups, only flies with full bloodmeals were selected for analysis and maintained for 4 days p.i. In the *Lu. longipalpis*-*L. mexicana* vector-parasite model the 4^th^ day of infection represents the population of parasites that have successfully survived bloodmeal digestion and defecation and are those that will colonize the rest of the fly for onward transmission^8^. The proportion of infected flies and midgut parasite loads revealed that lesions initiated with 100% *L. mexicana* metacyclics were significantly more infectious to *Lu. longipalpis* sandflies compared to those generated from various mixtures of infectious and non-infectious promastigotes (*P* < 0.005, Fig. 6c). Collectively, these data confirm that the composition of the transmitted dose is an important determinant of disease outcome and onward transmission.

Neutrophils and IL-1β expression are associated with enhanced pathology of cutaneous leishmaniasis^31–35^. To assess the contribution of non-metacyclics to lesion formation we employed a dermal air-pouch model of inflammation^13^. Air-pouches were inflated on the backs of mice and injected with either PBS or 5 × 10^2^ sandfly-derived *L. mexicana* metacyclics on their own (100%) or premixed with 5 × 10^2^ sandfly-derived nectomonads (50%). Cells migrating into the air-pouches were characterized by morphology, and the supernatants analysed for chemokines CCL3 and CXCL2 and the pro-inflammatory cytokine IL-1β by ELISA (Fig. 6d, e). We observed that more macrophages responded to air-pouches infected with 100% metacyclics compared to 50% metacyclics, with a corresponding higher secretion of the macrophage–attracting chemokine CCL3. Infections with nectomonads present resulted in more neutrophil than macrophage recruitment, although the numbers of both cell types were lower than their 100% metacyclic counterparts. To further investigate their association with natural infection, we determined the expression of these innate immune mediators in high dose bites (≥ 10^3^ promastigotes) from day 8 p.i. *L. mexicana*-infected flies, separated into either high (> 95%) or low (< 95%) proportions of metacyclics in the infectious bite (Fig. 6f). In accordance with the air-pouch model, low-quality transmission associated with a higher expression of CXCL2 and IL-1β, and high-quality transmission associated with a higher expression of CCL3. More recently, IL-1β activity and neutrophil recruitment in skin was shown to be promoted by the introduction of sandfly-associated bacteria with the bite^35^. Using expression of 16S rRNA as a proxy for the presence of bacteria associated with the skin (from the skin and/or the sandfly), we show that low-quality transmissions resulted in more bacteria compared to high-quality ones, and was associated with higher expression of IGF-1, promoted by regurgitation of infection-enhancing PSG from high-dose transmissions^36^. Collectively, these findings demonstrate that the proportion of metacyclic and non-metacyclic forms in the infective dose influence the magnitude of lesion severity and onward transmission to sandflies.

### Heterogeneity in dose increases *Leishmania* transmission potential

The potential epidemiological impact of transmission variability is made most transparent by accounting for it in calculations for the basic reproduction number (*R*_0_): a transmission metric that denotes the average number of secondary infections produced by a primary infection introduced into a susceptible population. In vector-borne disease systems it has been acknowledged for decades that some hosts are bitten more than others, and Dye and Hasibeder^37^ developed methods to account for this heterogeneity: *R*_*0*_ is inflated by the factor 1 + *α*, where *α* is the coefficient of variance squared. The coefficient of variance is calculated as the standard deviation for all observations in the sample divided by the mean. A comprehensive discussion of the theory behind this inflation factor, and its utility in analysing over-dispersed data, can be found here^38^.

In the current study, high variability was recorded for individual sand flies in the number of infective metacyclics they transmit in bites (Fig. 2a, d). We have previously shown that biting persistence increased linearly with metacyclic burden among sandflies^20^. Therefore, biting among the proportion of sandflies that are heavily infected with infective stages of parasite is intensified. Only with the quantitative methods presented in the current analysis is it possible to parameterize the coefficient of variance to allow for this inflation. The resulting effect on the *R*_*0*_ of *L*. *mexicana* is that it is increased more than 5-fold [1 + (9142/4416)^2^ = 5.3)] when this heterogeneity is incorporated, and for *L*. *infantum*, transmission potential is increased 6-fold [1 + (448/201)^2^ = 6.0)].

## Discussion

The composition of the dose of *Leishmania* promastigotes delivered by individual sandflies into the skin of a mammalian host has never been determined before. Here we design and validate a new approach to quantify and characterize *Leishmania* metacyclogenesis in sandflies and transmission to mice using RTqPCR of *sherp* gene expression. This advances existing qPCR methods that only quantify total numbers of *Leishmania* promastigotes^19,24,25^, as it allows the investigator to simultaneously assess the infectivity of sandflies and the quality of experimental transmission by determining the proportion of infectious metacyclics in the midgut and inoculated by each bite. Using this technique, we demonstrate that the majority (71–76%) of infected bites contained enriched doses of metacyclics (80–100%). However, a quarter (for *L. mexicana*) to a third (for *L. infantum*) of bites contained less than 75% metacyclics and for *L. mexicana* these were almost exclusively transmitted from flies harboring intermediary-large infections (10^4^–3 × 10^5^ parasites/midgut), accompanying the egestion of up to 35% of the midgut infection.

By analyzing the bites from flies as the infection progressed a window of optimal transmissibility opened midway through metacyclogenesis. This suggests that *Leishmania* transmission is more efficient from flies that have low-intermediary parasite burdens, which transmit lower doses but deliver bites more enriched for metacyclics. If this is the case, how can flies with immature infections (e.g., day 5 infections containing less than 10% metacyclics) transmit > 95% metacyclics? Previously, we have observed that metacyclics accumulate towards the ends of the PSG plug, in the anterior midgut and the stomodeal valve where transmission by regurgitation is more likely^8^. This points toward the possibility of an active form of selection taking place in the fly during transmission, enriching the bite for metacyclics - a hypothesis that remains to be explored in future studies.

In support of this, very large sandfly infections appeared to limit the size of the infectious dose but select for the deposition of metacyclics. In contrast, flies with intermediary infections tended to transmit larger doses of poorer quality, depositing larger proportions of their midgut infection, presumably through regurgitation of the PSG blockage and the parasites within. From this data, we extrapolate that for *L. mexicana*, ‘optimal’ transmission, containing high proportions of metacyclics, occurred when the fly had infections either below 5.4 × 10^3^ or above 3.86 × 10^5^ promastigotes per gut for doses of ≥ 80% metacyclics. A similar result was found when analyzing the correlates of high- and low-quality doses from infected flies fed once or multiple times, such that high-quality transmissions (> 95% metacyclics) resulted from a wide range of proportion or number of gut metacyclics, and flies with few gut metacyclics could also transmit a high-quality dose. This may also reflect the nature of the blockage in these infected midguts and the parasite’s interaction with it.

Proportionally, more non-metacyclics were transmitted by flies with large infections and they delivered the highest proportion of their midgut infection. Furthermore, blockage of the fly with PSG correlated closely with the dose quality of flies transmitting in their first bite, showing us that it is a strong selective force for the deposition of metacyclics. However, meta-analysis of multiple fed flies allowed to bite 10 times, suggests that lower PSG in the midgut post-feed reflects greater regurgitation of PSG during multiple feeding, resulting in metacyclic-enriched bites. This may be due to the fact that most of it is egested during low-quality previous transmissions where non-metacyclic forms (nectomonad and leptomonad promastigotes) appear to be immobilized within the PSG^8,11^. In later transmissions, when the PSG is depleted and/or solubilized in the midgut, it is likely that characteristics of the metacyclic promastigote, such as strong swimming, small body and long flagellum, could take over as determining factors in the composition of the dose.

In the present study, we observed that the parasite burden in the context of PSG amount in the sandfly midgut was a strong predictor of parasite dose and the number of transmitted metacyclics. However, this relationship broke down for the majority of our infected flies with mature intermediary-heavy infections, which passively regurgitated more non-metacyclics embedded within the PSG plug during their first bite. Nevertheless, this cohort of high-dose, low-quality transmitters are likely to be important to spread the infection since we have shown that these sandflies are more likely to deposit more infection-enhancing PSG^9,13^ and have increased feeding persistence, because of the higher amounts of PSG blocking their midgut^20^. Further, we may also expect these flies to deliver increasingly higher proportions of metacyclics with each successive bite as shown with our multiple bite experiments. Transmission heterogeneities such as these may have a large impact on *R*_*0*_ as they potentiate host contact and vector infectiousness^39,40^. By modelling the contribution of *Leishmania* dose heterogeneity and increased feeding persistence to transmission, we find that these flies can increase transmission potential by up to 6-fold. Inflated transmission potential derived from heterogeneous biting is more typically discussed in the context of hosts, whereby a small proportion of ‘attractive’ individuals are bitten more and contribute disproportionately to transmission^39–43^. Although mathematically analogous, being vector-centric, the heterogeneity in our analysis depicts a phenomenon that is distinct from these previous modelling studies.

Subclinical infections represent the majority of human and animal infections^44,45^. Our results show that low-dose, metacyclic-rich *L. mexicana* transmissions from the majority of infected *Lu. longipalpis* establish the host as an efficient reservoir of infection; similar to Kimblin et al^24^. In addition, we find that low-dose infections of mice that are metacyclic-rich generate minimal pathology yet establish a persistent population of parasites with high transmissibility back to sandflies. A recent study by Serafim and colleagues demonstrated that multiple bloodmeals resulted in larger infections with a higher proportion of metacyclics^46^. Importantly, they observed a 4-fold increase in lesion frequency on mice bitten by twice-fed *L. major*-infected *P. papatasi* compared to single-fed flies. The authors speculate that multiple bloodmeals are likely to result in a higher proportion of high-dose transmitters. Based on our results, we would predict that multiple bloodmeals would not only promote high doses but also the transmission of higher proportion of metacyclics. Taking our results and those of Serafim et al. together, the possibility of an enhanced presence of high-dose, metacyclic-rich transmitters following multiple bloodmeals may influence the epidemiology of leishmaniasis. This would result in more cases of disease with reduced severity, perhaps even asymptomatic infection, and result in more opportunities for new sandflies to pick up an infection. Recently, Doehl and colleagues found that outward transmission of subclinical *Leishmania donovani* infections in the skin of immunocompromised mice was best achieved if the distribution of parasites were patchy at both the macro-scale (over the body of the mouse) and micro-scale (within a patch); favoured by the pool-feeding nature of sandfly bloodfeeding^47^. Therefore, as sandflies tend to feed multiple times from hosts in close proximity^4,28,48^, which is likely to be exaggerated when the fly is infectious^20^, we would predict that heterogenous parasite transmission, acquisition, and the ability of multiple bloodmeals to enhance sandfly infectivity would combine to promote intense focal transmission.

Experimentally, the metacyclic-qPCR has provided new insight to *Leishmania* transmission and its impact on disease progression. Our findings indicate that heterogeneity in metacyclic promastigote exposure contributes considerably to heterogeneity in infection risk and variation in *Leishmania* transmission potential. Establishing the heterogeneity of the infectious inoculum and its effect on disease pathology will improve our understanding of the variability of natural transmission events.

## Methods

### Ethical statement for animal studies

All animal experiments were carried out in accordance with the UK Animal Scientific Procedure Act (ASPA) 1986, which transposes European Directive 2010/63/EU into UK national law.

The animal studies were approved by the UK home office in granting Project licence 70/8427 under the Animal Scientific Procedure Act and all protocols had undergone appropriate local ethical review procedures by the Animal welfare and Ethical Review Board (AWERB) of The London School of Hygiene and Tropical Medicine.

### *Leishmania* culture and morphology

*Leishmania mexicana* (MNYC/BZ/62/M379) or *L. infantum* (syn. *Leishmania chagasi*) (MHOM/BR/76/M4192) were cultured as previously described^8,17^. All cultures were initiated with 1 × 10^6^ skin lesion (*L. mexicana*) or splenic (*L. infantum*) amastigotes per ml from infected BALB/c mice. For *L. mexicana* and *L. infantum* nectomonad promastigotes were obtained from mid-logarithmic phase cultures (day 2 or 3) in M199 medium (Invitrogen, supplemented with 1% penicillin-streptomycin (v/v), 1 x BME vitamins (v/v), 10% heat-inactivated foetal calf serum (v/v)), pH 7.2 at 26 °C. *Leishmania infantum*, metacyclic promastigotes were purified from stationary phase M199 cultures (day 9–10) by differential centrifugation over a Ficoll gradient^49^. *Leishmania mexicana* metacyclics were obtained by passaging mid-log nectomonads into Grace’s insect culture medium (Invitrogen, supplemented as above), pH 5.5, 26 °C at 5 × 10^5^ per ml, harvested 7–8 days later and purified over Ficoll. Promastigotes were washed extensively in PBS before use and morphologically assessed through measurement of parasites from Giemsa-stained smears^8^. In addition, their sensitivity to complement killing was determined in vitro by exposure to fresh 5% pooled human serum for 30 min at 34 ^o^C, to ensure appropriate populations were used for real-time quantitative PCR (RTqPCR) and animal infections. Typically, cultured generated metacyclic promastigotes were 90–95% resistant to human serum. For infected sand fly midguts, formalin-fixed samples of parasites were counted using a Neubauer hemocytometer, and their developmental morphology assessed as above.

### Sandfly infection

Five-day-old *Lutzomyia longipalpis* (Jacobina strain) female sandflies were infected with *L. mexicana* or *L. infantum* amastigotes through an artificial membrane feeding system at a density of 2 × 10^6^ amastigotes per ml in heparinized, heat-inactivated pooled human blood. *L. mexicana* amastigotes were harvested from the rump lesions of female BALB/c mice, and *L. infantum* amastigotes were isolated from the spleens of female BALB/c mice^20^. Blood-fed flies were separated and maintained under a 12 h light:dark cycle at 26.5 °C, 80%–95% relative humidity, and supplied 25% (w/v) sucrose ad libitum. Flies were denied the opportunity to lay eggs to minimize post-oviposition mortality^20^, and all dissected flies with mature infections were observed to contain eggs.

### Experimental transmission

Age- and weight-matched female BALB/c mice were used as the source of the blood meal in each experiment. Mice were anesthetized by intraperitoneal (i.p.) injection of 30 μl Ketaset/Rompun mixture (100 mg kg^−1^ and 10 mg kg^−1^ body weight) and placed into a 25 cm3 netted cage; their bodies were screened with netting except for their ears, or in some experiments, their right infected leg. Care was taken to keep the same orientation and position of the mice for each exposure. For infected bites to the ear, flies were released into the cage singly. A sandfly was removed and recorded as a “no-feed” when 5 min after release had elapsed without it initiating a feed. In the majority of experiments, flies that began feeding within this time were allowed to feed for 5 min before removal for analysis. In multiple bite experiments flies were allowed 45 s before they were interrupted by gently brushing the antennae^20^, forcing them to fully retract their mouthparts from the skin before initiating a new bite at a different site. Flies took their first bite on the right ear by covering the left ear with micropore surgical tape. After this bite the fly was removed and the surgical tape was swapped over to cover the right ear. The fly was then reintroduced into the cage and encouraged to take multiple feeds on the left ear. Repeating the procedure of removing the fly and swapping the surgical tape allowed the 5th and 10th bites to be taken from the right ear. For an overview of the process, please see Supplementary Figure 8. If a fly approached the same position of previous bites on the right ear their antennae were gently brushed to encourage them to bite another part of the ear. For all bite experiments, a detailed plan of the position of each bite was recorded and bites in the same position were not processed. At the end of the experiment, sandflies were transferred to a glass vial, knocked down on ice, and their midguts dissected to assess distension by measuring the diameter of the stomodeal valve. Guts were then homogenized in PBS to quantify the infection by direct counting, measure haemoglobin levels using Drabkin’s reagent (Sigma-Aldrich, UK)^8^ and determine the relative quantity of PSG by semi-quantitative dot blot using LT15, a monoclonal antibody, that recognises the galactose-phosphate-mannose repeats in *Leishmania* phosphoglycans^9,10^. To assess the transmissibility of ear lesions to uninfected sandflies (xenodiagnosis) 250 day 2–4 female sugarfed flies, starved overnight, were allowed to feed on groups of five anaesthetised, infected BALB/c mice screened with netting and micropore tape except for their infected right ear. Fully engorged flies that bloodfed on the infected lesion were transferred to another cage and maintained for 4 days, as above, before dissecting their midgut to assess their infection.

### Infection of mice

Six- to 8-week-old female BALB/c mice were infected either by intradermal (i.d.) injection of 2.5 × 10^2^–1 × 10^3^ metacyclic promastigotes or nectomonad promastigotes in 10 μl via insulin needles into the dorsal surface of the right ear, as indicated. Lesion development was monitored by measuring the diameter of the swelling or lesion with Vernier callipers. At the end of experiments, mice were humanely euthanized, and parasite burdens in the ear determined by direct counting via hemocytometer^13^. All procedures involving animals were approved by a local Animal Welfare Committee and performed in accordance with United Kingdom Government (Home Office) and EC regulations. The distribution of values did not show evidence of non-normality using the Shapiro-Wilk test and therefore parametric analysis was performed (*t* tests). The null hypothesis was rejected if *P* < 0.05.

### Infection of dermal air-pouches

A concentration of 3 ml of sterile air was injected i.d. into the backs of shaved BALB/c mice to inflate an air-pouch. Into each air-pouch a total of 1 × 10^3^ *L*. *mexicana* promastigotes of varying proportions of metacyclics to non-metacyclics were injected in a total of 150 μl endotoxin-free PBS using a 27-gauge needle. At 48 h post-injection the cells from the air-pouch were recovered using a 5 ml ice-cold medium cavity lavage. Supernatant from the first 0.5 ml of the lavage was retained for cytokine and chemokine analysis and the cells combined with the following 4.5 ml of the lavage. The cells were concentrated to 0.5 ml by centrifugation (1800 rpm, 5 min) and live cells counted by diluting in trypan blue dye using a Neubauer improved haemocytometer. To determine the proportion of neutrophils and macrophage/monocytes by morphology, cells were concentrated on to slides using a Shandon cytospin 2 (500 rpm, 5 min) and stained with 10% (v/v) Giemsa’s stain.

### Tissue preparation and RNA extraction

During transmission, a record was made of the feeding position of each fly on each ear. Following exposure, bites were recovered by means of a 2 mm diameter punch biopsy centered on the bite site (presenting as a small erythematous dot) and rapidly frozen on dry ice. This minimized and standardized the amount of host skin processed to improve detection of rarer parasite transcripts. Ear biopsies were stored at −80 °C until required. Ear tissue was fragmented using a MiniBeadBeater (1 min, 5000 rpm) in 500 µl of lysis buffer with Precellys ceramic 2.8 mm beads, as previously described^50^. Dissected sandfly midguts were directly added to 250 μl lysis buffer and dispersed by drawing up and down in a pipette. RNA isolation was performed on the homogenate with the RNeasy Plus Mini kit (Qiagen, Courtaboeuf, France), according to the manufacturer’s instructions and eluted into 30 µl of RNase free water. RNA quality and quantity were determined using Nanodrop (ThermoFisher Scientific).

### Oligonucleotide primers and real-time quantitative polymerase chain reaction

A panel of parasite genes were chosen according to the different quantity of transcripts during the life cycle of *Leishmania* (Supplementary Table 1). qPCR was performed and analysed as previously described^36^. *Nono* and *l19* were selected as the most stable reference genes for the BALB/c ears^36,50^ and *MSLIST6001* was selected for *Lu. longipalpis* midguts^51^. Primer sequences are shown in the Supplementary Table 9.

### Determination of *L. mexicana* and *L. infantum* transcripts by RTqPCR in samples

Serial 10-fold dilutions of *L. mexicana* and *L. infantum* metacyclic promastigotes (10^6^ to 10^0^) were combined with nectomonad promastigotes (95%, 85%, 75% metacyclics) with a quarter of a mouse ear from naïve BALB/c mice, or with an uninfected sand fly midgut. The *Leishmania* gene target (*ssrRNA*) was used to quantify the number of parasites^52^ and a linear regression for each standard curve was determined using the number of *Leishmania* parasites against the Ct values of *ssrRNA*^50^. The *Leishmania* metacyclic gene target (*sherp*) was used to quantify the number of metacyclic promastigotes. A linear regression for each standard curve was determined (i.e. number of *Leishmania* parasites vs. Ct of *sherp*) and the ratio metacyclic number/non-metacyclic number was used to determine the proportion of metacyclic promastigotes (Supplementary Figure 4). Transcripts were determined from whole infected sandfly midguts or from different promastigote stages harvested from pools of 50–200 infected sandfly guts. To obtain enriched populations of the different promastigote stages, guts were sampled at various times based on previous experience^8,20^ (stated in Fig. 1 legend). 1 × 10^7^ cultured cells and 7 × 10^5^–1 × 10^6^ sandfly derived cells were tested per replicate after they had been washed three times in ice cold PBS, flash frozen in liquid nitrogen and stored at −80 °C until required.

### Determination of bacterial and host transcripts by RTqPCR in skin

A 2 mm diameter biopsies of skin were used to sample bites as described above. For a number of mice, cytokine, chemokine and bacterial transcripts were determined using RTqPCR in parallel to *ssrRNA* and *sherp* analysis. Skin biopsies from the opposite, naïve ear were used as the negative controls. We used *nono* and *l19* genes as housekeeping genes^36^. Primer information can be found in Supplementary Table 7.

### Luminex of dermal air-pouch supernatants

The first 0.5 ml of dermal air-pouch supernatants were assayed for CCL3, CXCL2 and IL-1β using a Bio-Plex Mouse cytokine assay (Biorad) according to the manufacturer’s protocols and quantified using a Luminex® 200 System (Luminexcorp)^36^.

### Statistical analyses

The *p* values were determined with GraphPad Prism software version 5.0. As the data was not normal (assessed by a Shapiro–Wilk normality test), the Mann–Whitney unpaired t-test was used to test the value statistical significance between groups for most experiments. Results from sequential bites from the same infected sandfly was analysed using a Wilcoxon paired test (*P < 0.05, **P < 0.005, ***P < 0.0005). All statistical tests were two-tailed. Linear correlation coefficients were generated with GraphPad Prism by Spearman-rank correlation to assess the association of parameters of sand fly infection on the composition of the dose.

### Reporting Summary

Further information on experimental design is available in the Nature Research Reporting Summary linked to this article.

## Supplementary information

Supplementary Information

Reporting Summary

## Data Availability

All data generated or analysed during this study are included in this published article (and its supplementary information files).
