# Supplementary material for: Quantifying Leishmania Metacyclic Promastigotes from Individual Sandfly Bites Reveals the Efficiency of Vector Transmission
Source: Commun Biol. 2019 Feb 28;2:84. doi: 10.1038/s42003-019-0323-8 (PMC6395631; doi:10.1038/s42003-019-0323-8)
Supplement: Supplementary file 1 — Supplementary Information [file 42003_2019_323_MOESM1_ESM.pdf]

Supplementary Information

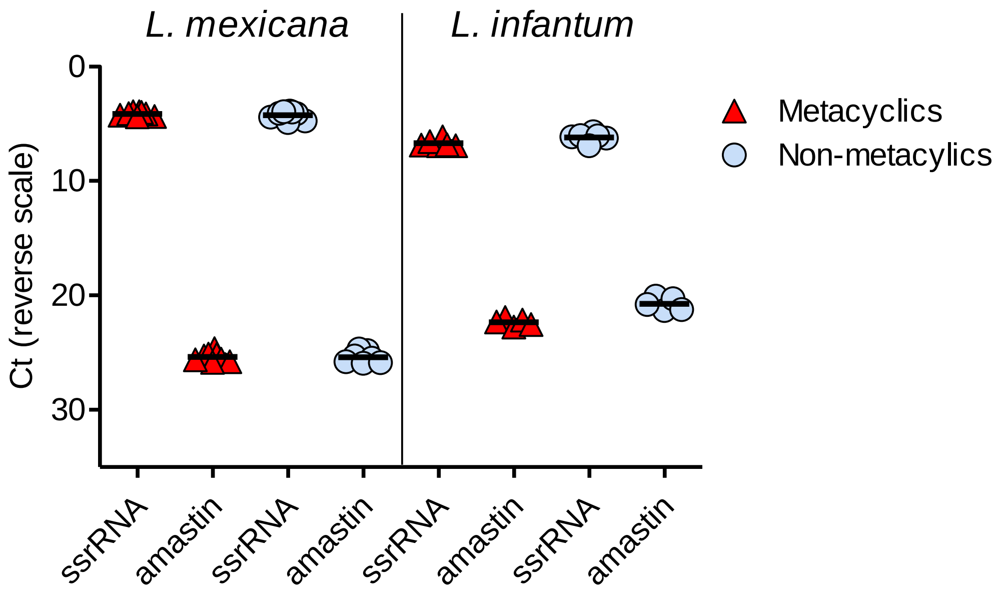

Supplementary Figure 1. *Leishmania mexicana* and *L. infantum ssrRNA* cycle-threshold (Ct) values of *ssrRNA* and *amastin* targets for metacyclic and non-metacyclic (nectomonad) promastigotes. Data shown are representative of 3 independent experiments. Solid lines represent means ±1 s.e.m.

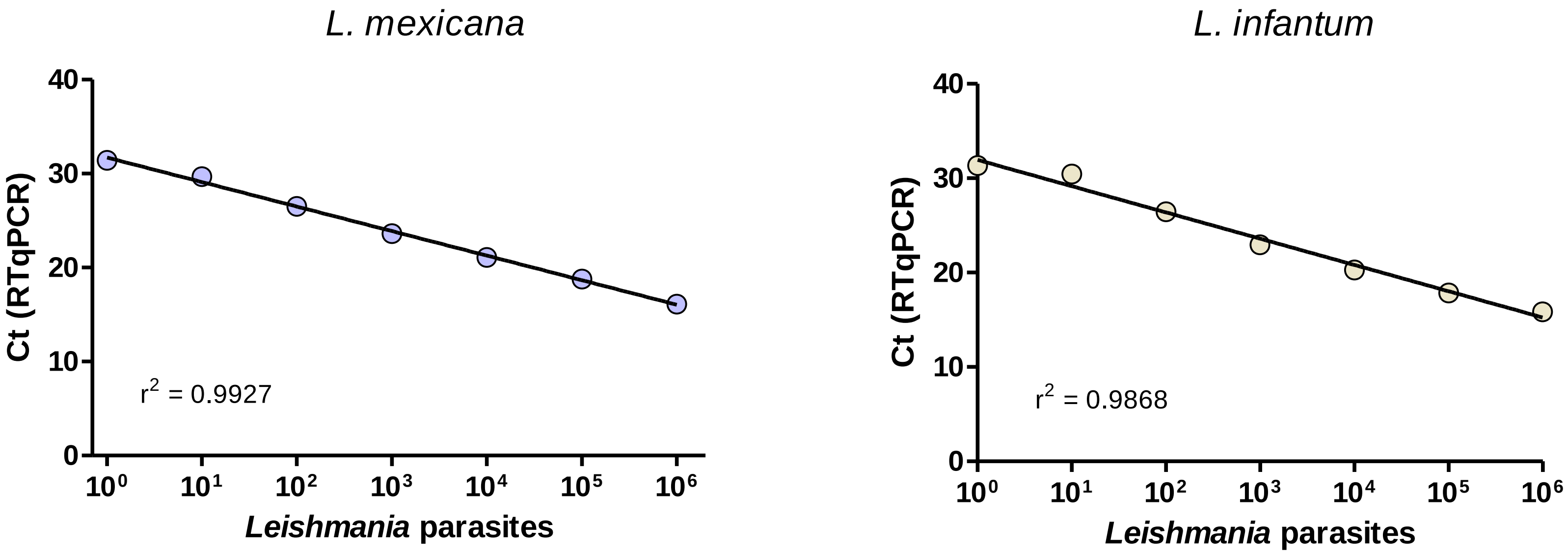

Supplementary Figure 2. *Leishmania mexicana* and *L. infantum ssrRNA* RTqPCR standard curves. Mean cycle-threshold (Ct) values for *ssrRNA* target and intra-assay SDs of a dilution series of non-metacyclic promastigotes. Results pooled from 5 independent repeat runs with each parasite dilution sampled in triplicate. Intra-coefficient of variance (CV) calculated from raw Ct values.

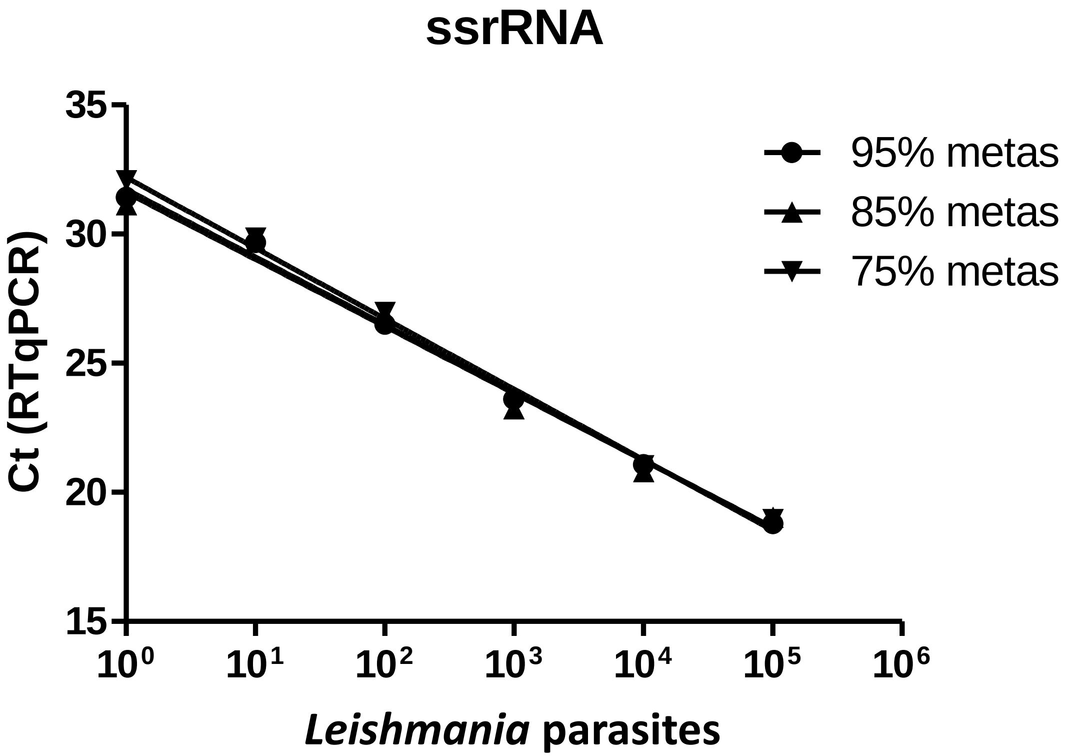

Supplementary Figure 3. ***Leishmania mexicana ssrRNA*** **RTqPCR standard curves.** Mean cycle-threshold (Ct) values for *ssrRNA* target and intra-assay SDs of a dilution series of metacyclic and non-metacyclic promastigotes over a range of cell numbers (1- 1 x 10^5) and ratios (95%-75% metacyclics). Results pooled from 5 independent repeat runs with each parasite dilution sampled in triplicate. Intra-coefficient of variance (CV) calculated from raw Ct values.

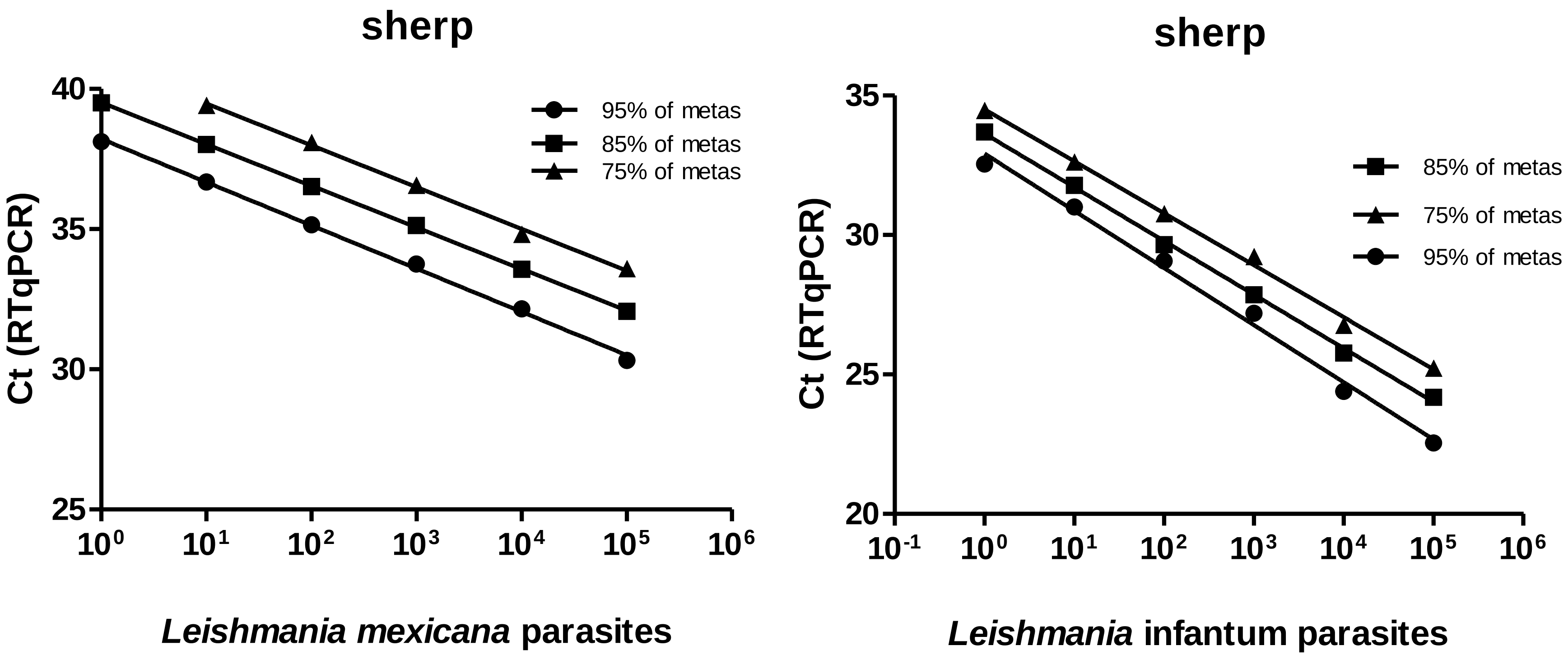

Supplementary Figure 4. ***Leishmania mexicana*** **and** ***L. infantum sherp*** **RTqPCR standard curves.** Mean cycle-threshold (Ct) values for ssrRNA target and intra-assay SDs of mouse ear tissue spiked with a dilution series of non-metacyclic promastigotes with different proportions of metacyclics/non-metacyclics. Results pooled from 5 independent repeat runs with each parasite dilution and % metacyclics sampled in triplicate.

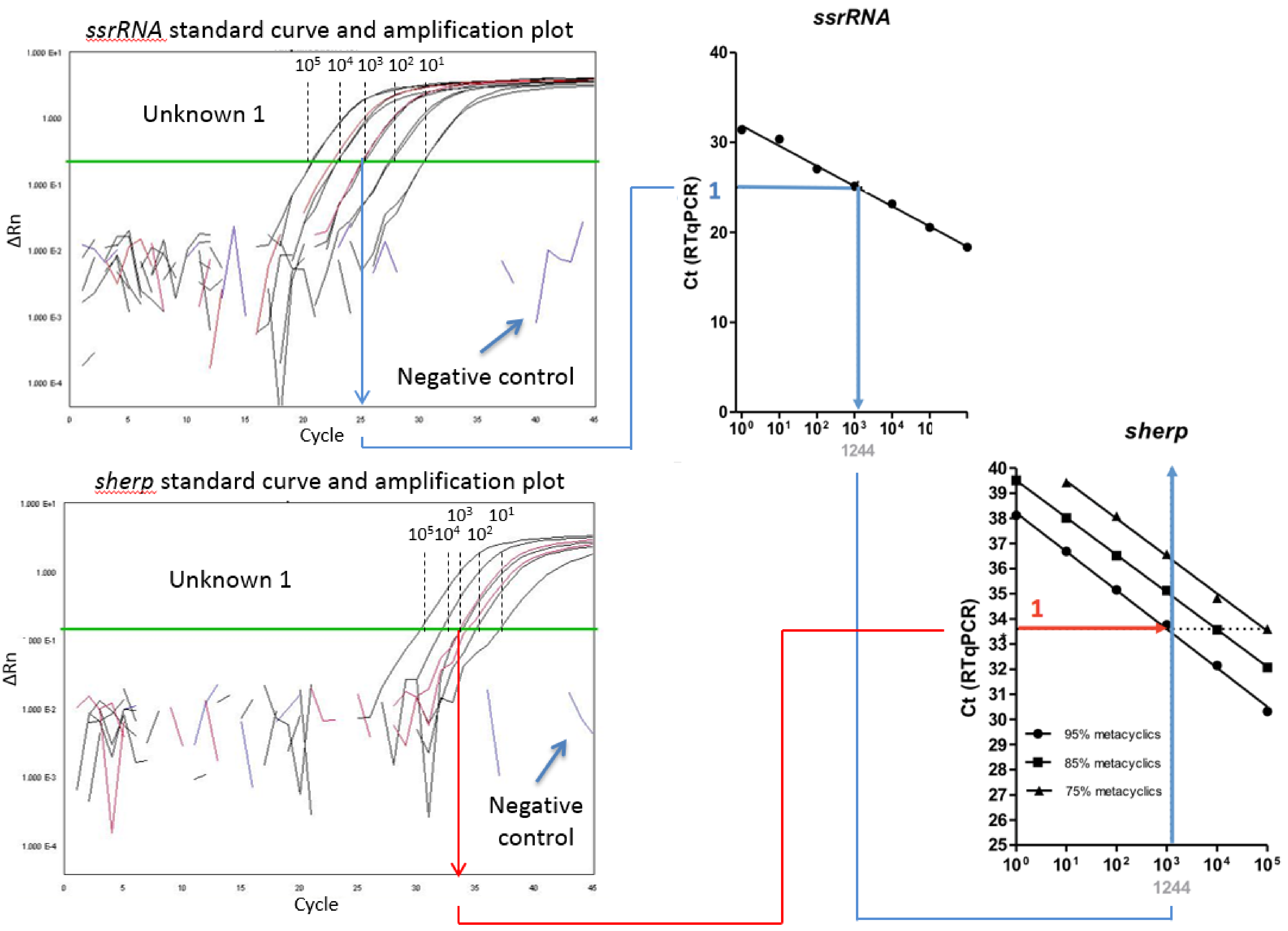

ssrRNA standard curve and amplification plot
Unknown 1
Negative control
ΔRn
Cycle
sherp standard curve and amplification plot
Unknown 1
Negative control
ΔRn
Cycle
ssrRNA
Ct (RTqPCR)
1244
sherp
Ct (RTqPCR)
95% metacyclics
85% metacyclics
75% metacyclics
1244

Supplementary Figure 5. Representative plots of RTqPCR for the quantitation of *Leishmania mexicana* in mouse ears bitten by individual infected sandflies. (*Left, top and bottom*) *ssrRNA* and *sherp* reaction signals ΔRn vs. cycle number (Ct value) co-extracted from known numbers of *L. mexicana* promastigotes with uninfected mouse ear tissue, plus a reaction produced by RNA extracted from an ear, exposed to a single infected fly (reactions for 95% metacyclics shown as representative curves). (*Right*, *top*) Standard curve generated by plotting *ssrRNA* Ct value vs. the log-transformed parasite numbers and the extrapolation of the parasite number present in the unknown sample. (*Right, bottom*) Standard curves generated by plotting Ct value of *sherp* expression vs. the log-transformed parasite number and the extrapolation of the parasite numbers present in the unknown sample against the Ct value for *sherp* expression in these samples. *Sherp* standard curves combined different proportions of culture-purified metacyclics with early log phase cultured non-metacyclic promastigotes (day 3, nectomonad promastigotes) over a wide a range of cells (1-1 x 10^6 promastigotes), with uninfected mouse ear tissue (Supplementary Table 4). Separate standard curves were generated with the inclusion of an uninfected sandfly midgut instead of mouse ear to estimate the infection load and proportion of metacyclics in infected flies. Plots were also generated for *L. infantum*. Data from this example is shown in Supplementary Table 5.

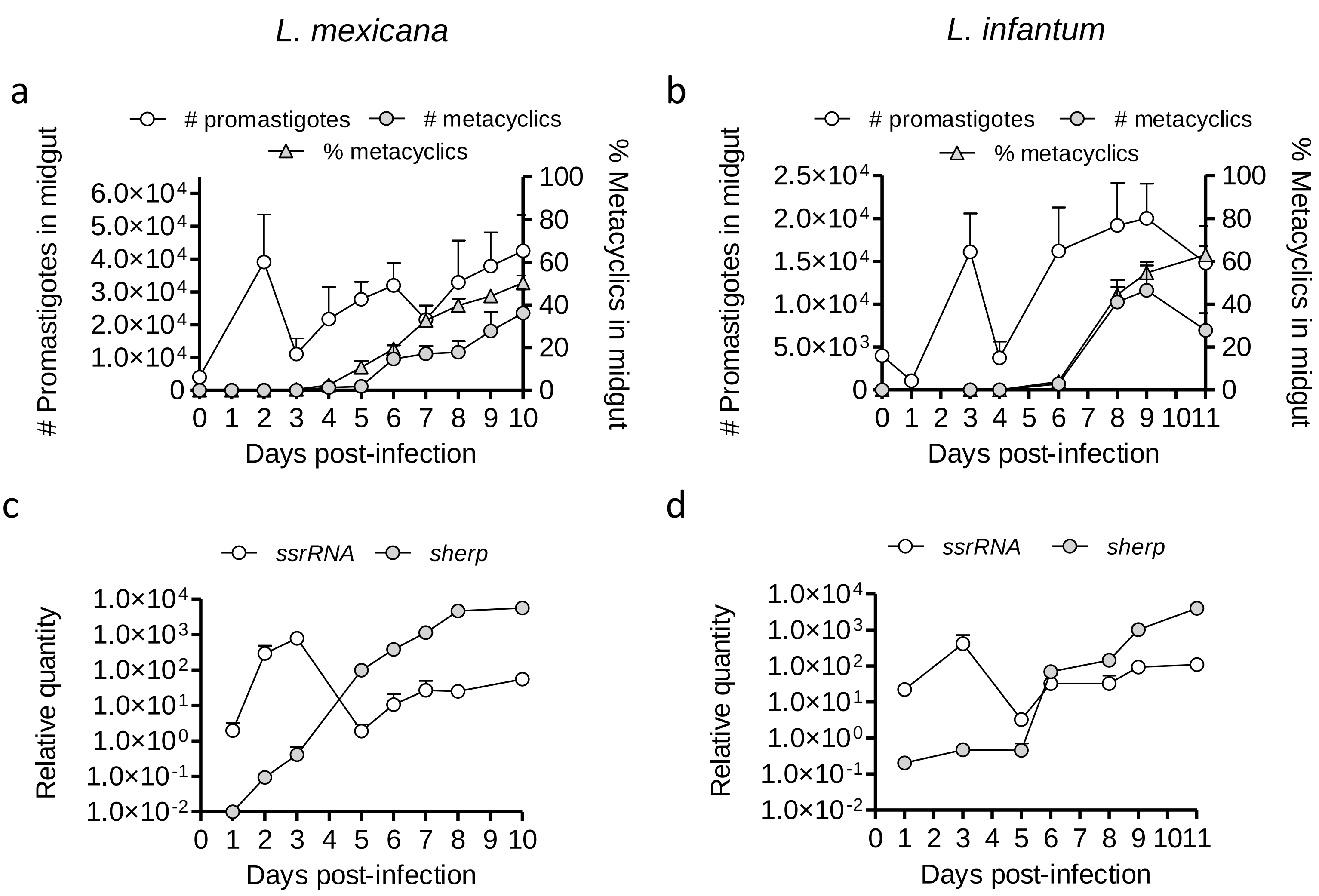

Supplementary Figure 6. Validation of *sherp* RTqPCR to measure metacyclogenesis in infected sandflies. (**a** and **b**) Infection dynamics of *L. mexicana* (**a**) and *L. infantum* (**b**) in *Lu. longipalpis* determined by microscopy. 10 flies were sampled daily. (**c** and **d**) Expression levels of *ssrRNA* and *sherp* in *L. mexicana* (**c**) and *L. infantum* (**d**) infected flies. 12 flies/day were sampled. Data represents the means ± 1 s.e.m.

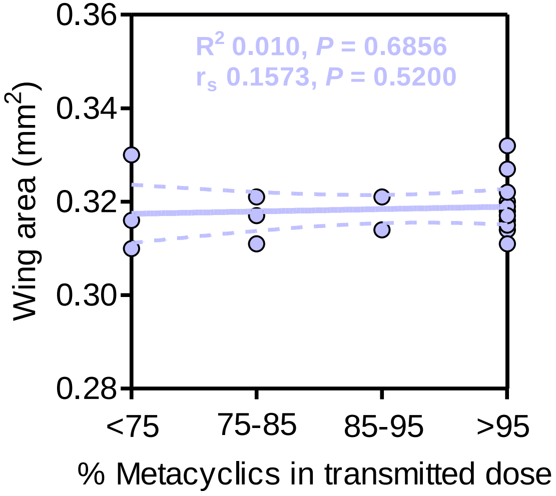

Supplementary Figure 7. Correlation between sandfly body size and metacyclic composition of the dose using wing area as a proxy for sandfly (pre-bloodfed) body size. Data is pooled from 4 independent experiments. Solid lines represent linear regression line of best fit and dotted lines represent the 95% confidence intervals. Correlation coefficients were generated by Spearman-rank correlation.

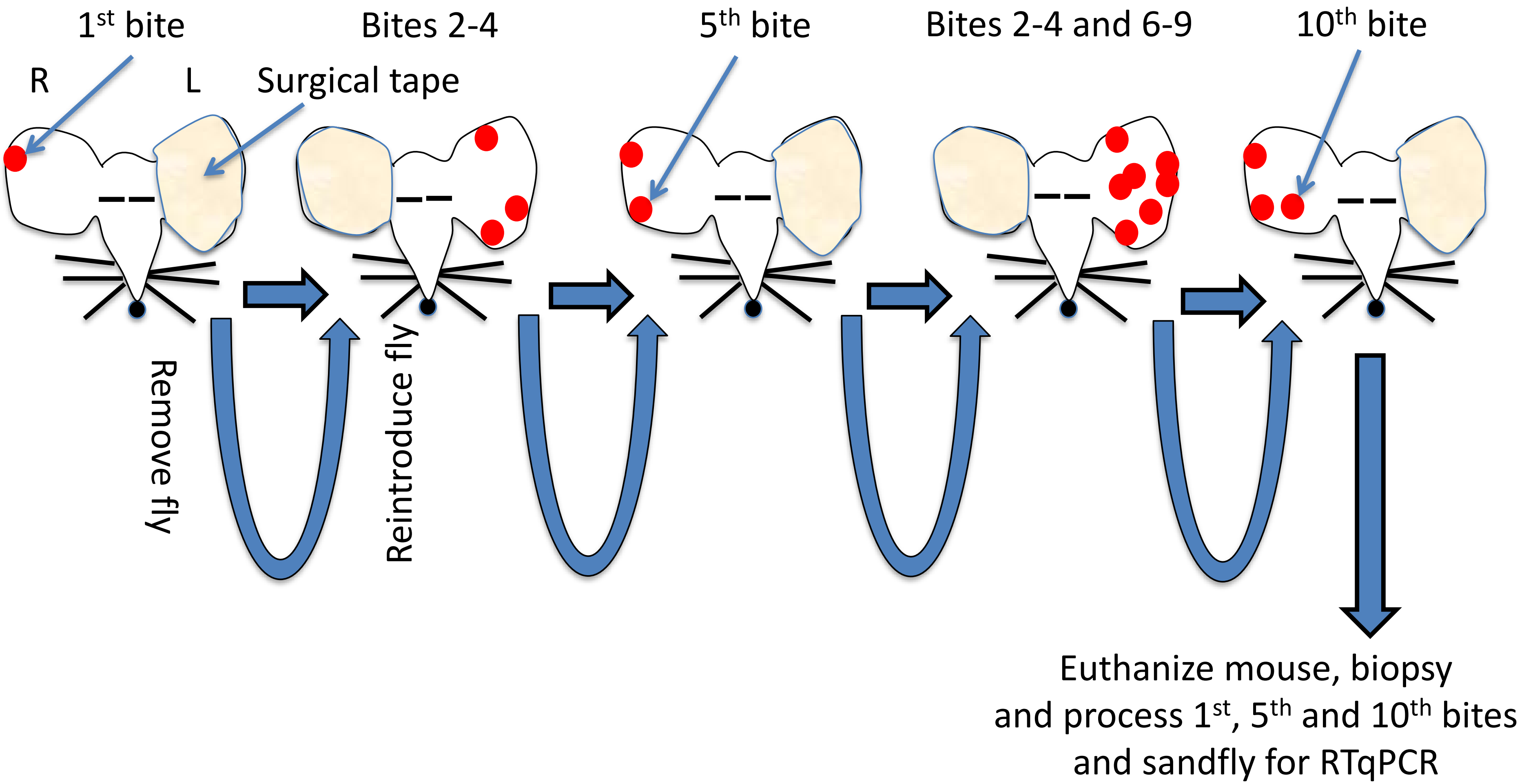

Supplementary Figure 8. Schematic of multiple bite methodology. Flies took their first bite on the right ear (R) of an anaesthetized BALB/c mouse by covering the left ear (L) with micropore surgical tape. After this bite the fly was removed and the surgical tape was swapped over to cover the right ear. The fly was then reintroduced into the cage and encouraged to take multiple feeds on the left ear. Repeating the procedure of removing the fly and swapping the surgical tape allowed the 5th and 10th bites to be taken from the right ear. If a fly approached the same position of previous bites on the right ear their antennae were gently brushed to encourage them to bite another part of the ear. For all bite experiments, a detailed plan of the position of each bite was recorded and bites in the same position were not processed.

Target	Gene	Amastigotes	Non-metacyclic promastigotes	Metacyclic promastigotes	Known functions	References
**Amastin**	Amastin	*L. infantum* +++<br>*L. mexicana* +++	*L. infantum* +<br>*L. mexicana* +	*L. infantum* +<br>*L. mexicana* +	Surface protein- hypothetical role in cytoplasmic pH regulation	Wu, 2000[1]; Nasereddin, 2010[2]; Rochette, 2005[3]; Jackson, 2010[4]; Real, 2013[5]
**Meta**	Meta	*L. infantum* +<br>*L. mexicana* +	*L. infantum* ++<br>*L. mexicana* ++	*L. infantum* +++<br>*L. mexicana* +++	Role in metacyclogenesis and virulence in hosts	Ramos, 2004[6]; Ramos, 2011[7]; Uliana, 1999[8]; Berberich, 1998[9]
**Small hydrophilic ER-associated protein**	Small hydrophilic ER-associated protein	*L. infantum* +<br>*L. mexicana* +	*L. infantum* ++<br>*L. mexicana* ++	*L. infantum* +++<br>*L. mexicana* +++	Peripheral membrane protein - Role in macrophage infection	Moore, 2011[10]; Ouakad, 2011[11]; Depledge, 2010[12]
**Hydrophilic acylated surface protein**	Hydrophilic acylated surface protein	*L. infantum* +<br>*L. mexicana* +++	*L. infantum* ++<br>*L. mexicana* ++	*L. infantum* +++<br>*L. mexicana* +++	Surface protein -Role in parasite transmission by sand fly	McKean, 2001[13]; Depledge, 2010[12]; Alce, 1999[14]
**MSP, leishmanolysin**	MSP, leishmanolysin	*L. infantum* ++<br>*L. mexicana* +	*L. infantum* ++<br>*L. mexicana* +	*L. infantum* +++<br>L. mexicana +++	Surface protease- Role in parasite multiplication, protection from complement, parasite attachment and virulence in macrophages	Frommel, 1990[15] ; Halle, 2009[16]; Medina-Acosta, 1993[17]; Yao, 2009[18]
**small subunit ribosomal RNA**	small subunit ribosomal RNA	*L. infantum* +++<br>*L. mexicana* +++	*L. infantum* +++<br>*L. mexicana* +++	*L. infantum* +++<br>*L. mexicana* +++	Directs information flow during protein synthesis	de La Llave, 2011[19]; Cavalcanti, 2015[20]
**Beta-tubulin**	Beta-tubulin	*L. infantum* +<br>*L. mexicana* +	*L. infantum* ++<br>*L. mexicana* ++	*L. infantum* +++<br>*L. mexicana* +++	Cytoskeletal protein	Fong, 1984[21]; Bellatin, 2002[22]; Jackson, 2006[23]; Bhaumik, 1991[24]

Supplementary Table 1. Transcripts associated with *Leishmania* metacyclic promastigotes. Relative expression level in the different life-cycle stages of *Leishmania* range from low (+), high (++) to very high (+++). Generated from published data.

Leishmania species	Mean *ssrRNA* Ct values ± 1 S.D. (Intra-assay CV) with the following total number of parasites per reaction:						
	1,000,000	100,000	10,000	1,000	100	10	1
Leishmania mexicana	16.12 ± 0.197 (1.22%)	18.80 ± 0.190 (1.011%)	21.08 ± 0.124 (0.588%)	23.60 ± 0.082 (0.347%)	26.51 ± 0.012 (0.045%)	29.68 ± 0.101 (0.340%)	31.42 ± 0.110 (0.350%)
Leishmania infantum	15.44 ± 0.167 (1.082%)	17.84 ± 0.137 (0.768%)	20.30 ± 0.205 (1.010%)	22.96 ± 0.170 (0.740%)	26.45 ± 0.198 (0.749%)	30.46 ± 0.299 (0.982%)	31.35 ± 0.163 (0.520%)

Supplementary Table 2. *Leishmania mexicana* and *L. infantum ssrRNA* RTqPCR standard curves. Mean cycle-threshold (Ct) values for *ssrRNA* target and intra-assay SDs of a dilution series of non-metacyclic promastigotes. Results pooled from 5 independent repeat runs with each parasite dilution sampled in triplicate. Intra-coefficient of variance (CV) calculated from raw Ct values. This table accompanies Supplementary Figure 2.

L. mexicana	Mean *ssrRNA* Ct ± 1 S.D.		
	95% metas	85% metas	75% metas
r^2	0.9954	0.9869	0.9931
Y intercept	31.74	31.62	32.20
100,000	18.80 ± 0.190	19.05 ± 0.133	18.96 ± 0.117
10,000	21.08 ± 0.124	20.81 ± 0.178	21.04 ± 0.108
1,000	23.60 ± 0.082	23.24 ± 0.109	23.33 ± 0.124
100	26.51 ± 0.012	26.86 ± 0.150	26.97 ± 0.140
10	29.68 ± 0.101	29.70 ± 0.131	29.85 ± 0.152
1	31.42 ± 0.110	31.15 ± 0.123	32.07 ± 0.114

Supplementary Table 3. *Leishmania mexicana ssrRNA* RTqPCR standard curves. Mean cycle-threshold (Ct) values for *ssrRNA* target and intra-assay SDs of a dilution series of metacyclic and non-metacyclic promastigotes over a range of cell numbers (1- 1 x 10^5) and ratios (95%-75% metacyclics). Results pooled from 5 independent repeat runs with each parasite dilution sampled in triplicate. Intra-coefficient of variance (CV) calculated from raw Ct values. This table accompanies Supplementary Figure 3.

Total number of parasites	Mean *sherp* Ct ± 1 S.D. (Intra-assay CV)			Total number of parasites	Mean *sherp* Ct ± 1 S.D. (intra-assay CV)		
	Leishmania mexicana				*Leishmania infantum*		
	95% metacyclics	85% metacyclics	75% metacyclics		95% metacyclics	85% metacyclics	75% metacyclics
100,000	30.32 ± 0.099 (0.327%)	32.07 ± 0.141 (0.44%)	33.60 ± 0.174 (0.518%)	100,000	22.54 ± 0.015 (0.067%)	24.18 ± 0.073 (0.302%)	25.23 ± 0.068 (0.270%)
10,000	32.15 ± 0.046 (0.143%)	33.57 ± 0.139 (0.414%)	34.82 ± 0.209 (0.6%)	10,000	24.40 ± 0.147 (0.602%)	25.77 ± 0.030 (0.116%)	26.77 ± 0.018 (0.067%)
1,000	33.76 ± 0.186 (0.551%)	35.15 ± 0.223 (0.634%)	36.57 ± 0.179 (0.489%)	1000	27.19 ± 0.085 (0.313%)	27.86 ± 0.119 (0.427%)	29.23 ± 0.064 (0.219%)
100	35.13 ± 0.268 (0.763%)	36.52 ± 0.177 (0.485%)	38.09 ± 0.096 (0.252%)	100	29.07 ± 0.213 (0.733%)	29.66 ± 0.127 (0.428%)	30.77 ± 0.063 (0.205%)
10	36.68 ± 0.193 (0.526%)	38.02 ± 0.168 (0.442%)	39.42 ± 0.187 (0.474%)	10	31.01 ± 0.079 (0.255%)	31.78 ± 0.068 (0.214%)	32.62 ± 0.162 (0.497%)
1	38.10 ± 0.234 (0.614%)	39.51 ± 0.238 (0.602%)	ND[a]	1	32.55 ± 0.244 (0.750%)	33.69 ± 0.169 (0.502%)	34.47 ± 0.198 (0.574%)
Correlation coefficient r^2	0.9980	0.9998	0.9973	Correlation coefficient r^2	0.9931	0.9988	0.9970
Y-intercept	38.22	39.51	40.97	Y-intercept	32.92	33.64	34.51

Supplementary Table 4. *Leishmania mexicana* and *L. infantum sherp* RTqPCR standard curves. Mean cycle-threshold (Ct) values for ssrRNA target and intra-assay SDs of mouse ear tissue spiked with a dilution series of non-metacyclic promastigotes with different proportions of metacyclics/non-metacyclics. Results pooled from 5 independent repeat runs with each parasite dilution and % metacyclics sampled in triplicate. Intra-coefficient of variance (CV) calculated from raw Ct values. [a] ND, inconsistent quantification data (Ct value >40).

Dose-composition determination					
Sample	ssrRNA (Ct)	Total number of parasites	sherp (Ct)	% of metacyclics (extrapolated %)	Estimated number of metacyclics
1	25	1244	33.6	85-95% (95%)	1182

Supplementary Table 5. Example of RTqPCR for the quantitation of *Leishmania mexicana* in mouse ears bitten by individual infected sandflies. This table accompanies Supplementary Figure 5.

Dose from infected *Lutzomyia longipalpis* sandflies	*Leishmania infantum*	*Leishmania mexicana*
**Mean**	268	4111
**± 95 % C.I.**	105-430	2379-5843
**Geometric mean**	21	333
**± 95% C.I.**	11-38	191-579

Supplementary Table 6. Additional transmission data for Fig. 2a and d.

Dose characteristics	Cluster 1 sandflies	Cluster 2 sandflies	Cluster 3 sandflies	Cluster 4 sandflies
**Sample size**	9	14	8	4
**Geometric mean dose ± 95 % C.I.**	8.24×10^2<br>2.81×10^2-2.96×10^3	4.66×10^4<br>2.21×10^4-1.04×10^5	1.65×10^6<br>2.86×10^5-1.21×10^7	1.41×10^6<br>1.00×10^6-1.98×10^7
**Median dose**	8.02×10^2,	3.92×10^4	1.19×10^6	1.19×10^6
**Spearman's correlation**	0.952<br>($P = 0.0011$)	0.8821<br>($P < 0.0001$)	0.5<br>($P = 1.0$)	0.1905<br>($P = 0.6646$)
**Mean proportion of fly infection transmitted ± 95 % C.I.**	7.7%<br>4.12-14.55%	2.42%<br>1.27-4.64%	0.008%<br>0.002-0.035% (Clusters 3 and 4 combined)	
**Median proportion transmitted**	10.45%	1.94%	0.005%	

Supplementary Table 7. Additional transmission data for Fig. 2 g-i.

Day post-infection	n	Infection level: promastigotes/fly (mean ± s.e.m.)	% metacyclic promastigotes (mean ± s.e.m.)
**5**	40	1.092×10^4 ± 4.533×10^3	9.3 ± 2.7
**7**	40	1.926×10^4 ± 6.023×10^3	24.6 ± 6.5
**9**	40	2.263×10^4 ± 8.005×10^3	59 ± 6.7

Supplementary Table 8. Average infection profile of sandflies at each day p.i. indicated. 10 flies/day/experiment were sampled from the cohort of flies to be used for transmission. Data is pooled from 4 independent infections. Flies from these cohorts were used in Fig. 3, 4 and 5.

Target organism	Target gene (Leishmania species)	Primer sequences		Melting temperature ºC
		forward	reverse	
Leishmania	amastin (*L. infantum*)	GCGGTGGAGACGTGCTG	GTGTAGGTAGAGTTGTCGCAGTTGT	84.8
	amastin (*L. mexicana*)	ACGAGACGATGCAGCACA	TCCGCCACCTGCTGTAAT	84.4
	haspb (*L. infantum*)	GCGTGCTGATAACATCCATAAA	TATGGCCATCCTCCTTCG	85.5
	haspb (*L. mexicana*)	TCGACTTGCTTATCCCCATAGT	TGTTGCTTCCAGTGGTTGTATC	82.4
	gp63 (*L. infantum*)	GTCTCAGCCTTGGAAAGTGC	GTCGGAGAAGACGTTGAAGC	86.9
	gp63 (*L. mexicana*)	GACCGTCTGAGAGTCGGAAC	CAGGGCGGTGTAGTATCCAT	85.7
	Meta1/2 (*L. infantum*)	GTAAACCGCAAGCTCTCTGG	CGAGCTTGATGGTGTGTGTT	82.7
	Meta1/2 (*L. mexicana*)	GGAACGGTCCAGTACGAAAA	CACCACATTGAATCCGTCAG	84.3
	sherp (*L. infantum*)	CGACAATGCGCACAACA	CGCCGCTTATCTTGTCCT	82.6
	sherp (*L. mexicana*)	CTGCAGCCTTGTTGCTCA	ACCAGGAGACAAGGGACCA	82.9
	ssrRNA * (*L. infantum* and *L. mexicana*)	CCATGTCGGATTTGGT	CGAAACGGTAGCCTAGAG	80.8
	tubulin (*L. infantum*)	CCAAGGAGGTAGACGAGCAG	GGAGACGAGGTCGTTCATGT	84.8
	tubulin (*L. mexicana*)	GGCTTCCAGCTGTCTCACTC	GTACGGCTCCACAACGGTAT	86.9
**Mouse**	nono	AAAGCAGTTAGAACTCAGG	GGCCCATCCGTATCTC	81.9
	l19	TACTGCCAATGCTCGG	AACACATTCCCTTTGACC	80.9
	CXCL2	CACCAACCACCAGGCTACAG	GCCCTTGAGAGTGGCTATGAC	83.8
	CCL3	ACCACTGCCCTTGCTGTTC	TCTGCCGGTTTCTCTTAGTCAG	82.6
	IL-1beta	AGGCAGGCAGTATCAC	CACACCAGCAGGTTATC	80.6
	IGF1	TGTTGCTTCCGGAGCTGT	TTGGGCATGTCAGTGTGG	82.5
**Sandfly (*Lu. longipalpis*)**	MSLIST6001	AAAGGGTGCGAAGTTATTGC	GGGTGGGTTGGACATTCTAC	79.0
**Bacteria**	16S (V1-3)	CCTACGGGAGGCAGCAG	CCGTCAATTCCTTTRAGTTT	81.0

Supplementary Table 9. RTqPCR primer information. * de La Llave et al. 2011[19]

Supplementary References

1. Wu, Y., El Fakhry, Y., Sereno, D., Tamar, S. & Papadopoulou, B. A new developmentally regulated gene family in *Leishmania* amastigotes encoding a homolog of amastin surface proteins. *Mol. Biochem. Parasitol.* **110**, 345–357 (2000).

2. Nasereddin, A., Schweynoch, C., Schonian, G. & Jaffe, C. L. Characterization of *Leishmania* (*Leishmania*) *tropica* axenic amastigotes. *Acta Trop.* **113**, 72–79 (2010).

3. Rochette, A. *et al.* Characterization and developmental gene regulation of a large gene family encoding amastin surface proteins in *Leishmania spp*. *Mol. Biochem. Parasitol.* **140**, 205–220 (2005).

4. Jackson, A. P. The evolution of amastin surface glycoproteins in trypanosomatid parasites. *Mol. Biol. Evol.* **27**, 33–45 (2010).

5. Real, F. *et al.* The genome sequence of *Leishmania* (*Leishmania*) *amazonensis*: functional annotation and extended analysis of gene models. *DNA Res.* **20**, 567–581 (2013).

6. Ramos, C. S., Franco, F. A. L., Smith, D. F. & Uliana, S. R. B. Characterisation of a new *Leishmania* META gene and genomic analysis of the META cluster. *FEMS Microbiol. Lett.* **238**, 213–219 (2004).

7. Ramos, C. S. *et al. Leishmania amazonensis* META2 protein confers protection against heat shock and oxidative stress. *Exp. Parasitol.* **127**, 228–237 (2011).

8. Uliana, S. R., Goyal, N., Freymüller, E. & Smith, D. F. *Leishmania*: overexpression and comparative structural analysis of the stage-regulated meta 1 gene. *Exp. Parasitol.* **92**, 183–191 (1999).

9. Berberich, C., Marín, M., Ramírez, J. R., Muskus, C. & Vélez, I. D. The metacyclic stage-expressed meta-1 gene is conserved between Old and New World *Leishmania* species. *Mem. Inst. Oswaldo Cruz* **93**, 819–821 (1998).

10. Moore, B. *et al.* Structural basis of molecular recognition of the *Leishmania* small hydrophilic endoplasmic reticulum-associated protein (SHERP) at membrane surfaces. *J. Biol. Chem.* **286**, 9246–9256 (2011).

11. Ouakad, M. *et al.* Increased metacyclogenesis of antimony-resistant *Leishmania donovani* clinical lines. *Parasitology* **138**, 1392–1399 (2011).

12. Depledge, D. P. *et al. Leishmania*-specific surface antigens show sub-genus sequence variation and immune recognition. *PLoS Negl Trop Dis* **4**, e829 (2010).

13. McKean, P. G., Denny, P. W., Knuepfer, E., Keen, J. K. & Smith, D. F. Phenotypic changes associated with deletion and overexpression of a stage-regulated gene family in *Leishmania*. *Cell. Microbiol.* **3**, 511–523 (2001).

14. Alce, T. M., Gokool, S., McGhie, D., Stäger, S. & Smith, D. F. Expression of hydrophilic surface proteins in infective stages of *Leishmania donovani*. *Mol. Biochem. Parasitol.* **102**, 191–196 (1999).

15. Frommel, T. O., Button, L. L., Fujikura, Y. & McMaster, W. R. The major surface glycoprotein (GP63) is present in both life stages of *Leishmania*. *Mol. Biochem. Parasitol.* **38**, 25–32 (1990).

16. Hallé, M. *et al.* The *Leishmania* surface protease GP63 cleaves multiple intracellular proteins and actively participates in p38 mitogen-activated protein kinase inactivation. *J. Biol. Chem.* **284**, 6893–6908 (2009).

17. Medina-Acosta, E., Karess, R. E. & Russell, D. G. Structurally distinct genes for the surface protease of *Leishmania mexicana* are developmentally regulated. *Mol. Biochem. Parasitol.* **57**, 31–45 (1993).

18. Yao, C. Major surface protease of trypanosomatids: one size fits all? *Infect. Immun.* **78**, 22–31 (2010).

19. de La Llave, E. *et al.* A combined luciferase imaging and reverse transcription polymerase chain reaction assay for the study of *Leishmania* amastigote burden and correlated mouse tissue transcript fluctuations. *Cell. Microbiol.* **13**, 81–91 (2011).

20. Cavalcanti, A. S. *et al.* Parasite load induces progressive spleen architecture breakage and impairs cytokine mRNA expression in *Leishmania infantum*-naturally infected dogs. *PLoS ONE* **10**, e0123009 (2015).

21. Fong, D., Wallach, M., Keithly, J., Melera, P. W. & Chang, K. P. Differential expression of mRNAs for alpha- and beta-tubulin during differentiation of the parasitic protozoan *Leishmania mexicana*. *Proc. Natl. Acad. Sci. U.S.A.* **81**, 5782–5786 (1984).

22. Bellatin, J. A., Murray, A. S., Zhao, M. & McMaster, W. R. *Leishmania mexicana*: identification of genes that are preferentially expressed in amastigotes. *Exp. Parasitol.* **100**, 44–53 (2002).

23. Jackson, A. P., Vaughan, S. & Gull, K. Comparative genomics and concerted evolution of beta-tubulin paralogs in *Leishmania spp*. *BMC Genomics* **7**, 137 (2006).

24. Bhaumik, M., Das, S. & Adhya, S. Evidence for translational control of beta-tubulin synthesis during differentiation of *Leishmania donovani*. *Parasitology* **103 Pt 2**, 197–205 (1991).
