# Supplementary material for: Quantifying Leishmania Metacyclic Promastigotes from Individual Sandfly Bites Reveals the Efficiency of Vector Transmission
Source: Commun Biol. 2019 Feb 28;2:84. doi: 10.1038/s42003-019-0323-8 (PMC6395631; doi:10.1038/s42003-019-0323-8)
Supplement: Supplementary file 2 — Reporting Summary [file 42003_2019_323_MOESM2_ESM.pdf]

nature research

Corresponding author(s): Matthew Rogers

Last updated by author(s): Jan 3, 2019

Reporting Summary

Nature Research wishes to improve the reproducibility of the work that we publish. This form provides structure for consistency and transparency in reporting. For further information on Nature Research policies, see Authors & Referees and the Editorial Policy Checklist.

Statistics

For all statistical analyses, confirm that the following items are present in the figure legend, table legend, main text, or Methods section.

n/a	Confirmed	
☐	☒	The exact sample size (*n*) for each experimental group/condition, given as a discrete number and unit of measurement
☐	☒	A statement on whether measurements were taken from distinct samples or whether the same sample was measured repeatedly
☐	☒	The statistical test(s) used AND whether they are one- or two-sided<br>*Only common tests should be described solely by name; describe more complex techniques in the Methods section.*
☐	☒	A description of all covariates tested
☐	☒	A description of any assumptions or corrections, such as tests of normality and adjustment for multiple comparisons
☐	☒	A full description of the statistical parameters including central tendency (e.g. means) or other basic estimates (e.g. regression coefficient) AND variation (e.g. standard deviation) or associated estimates of uncertainty (e.g. confidence intervals)
☐	☒	For null hypothesis testing, the test statistic (e.g. *F*, *t*, *r*) with confidence intervals, effect sizes, degrees of freedom and *P* value noted<br>*Give P values as exact values whenever suitable.*
☒	☐	For Bayesian analysis, information on the choice of priors and Markov chain Monte Carlo settings
☒	☐	For hierarchical and complex designs, identification of the appropriate level for tests and full reporting of outcomes
☒	☐	Estimates of effect sizes (e.g. Cohen's *d*, Pearson's *r*), indicating how they were calculated

Our web collection on statistics for biologists contains articles on many of the points above.

Software and code

Policy information about availability of computer code

Data collection	Cycle threshold values (Ct) were determined using the Applied Biosystems SDS2.4 Software. Ct values were input into Biogazelle qBasePLUS software. For normalisation calculations, candidate control genes were tested with geNorm and Normfinder programmes. Luminex microspheres were quantified using a Luminex® 100 System (Luminexcorp).
Data analysis	Data was analysed with GraphPad Prism software version 5.0. R version 3.4.1 with the Mclust package was used for the Gaussian mixture model-based cluster analysis.

For manuscripts utilizing custom algorithms or software that are central to the research but not yet described in published literature, software must be made available to editors/reviewers. We strongly encourage code deposition in a community repository (e.g. GitHub). See the Nature Research guidelines for submitting code & software for further information.

Data

Policy information about availability of data

All manuscripts must include a data availability statement. This statement should provide the following information, where applicable:

- Accession codes, unique identifiers, or web links for publicly available datasets
- A list of figures that have associated raw data
- A description of any restrictions on data availability

All data generated or analysed during this study are included in this published article (and its supplementary information files).

Field-specific reporting

Please select the one below that is the best fit for your research. If you are not sure, read the appropriate sections before making your selection.

☒ Life sciences ☐ Behavioural & social sciences ☐ Ecological, evolutionary & environmental sciences

For a reference copy of the document with all sections, see nature.com/documents/nr-reporting-summary-flat.pdf

Life sciences study design

All studies must disclose on these points even when the disclosure is negative.

Sample size	The sample size of analysed bites was based on a previous study (Kimblin et al. 2008, PNAS).<br>The sample sizes used in most infection experiments will be 6 mice per group, maintained for 80 days. Our previous studies (Rogers et al. 2009, PLoS Pathogens) have shown this is sufficient to show a 30% difference in parasite load with a power of 80% and at 95% confidence limits.
Data exclusions	No data were excluded from the analyses.
Replication	Experiments analysing the composition of the infectious dose from individual infected sandflies were obtained by pooling the results from 4 independent replicates. Experiments investigating the immunological consequence of dose composition were obtained from a representative experiment of 3 replicates.
Randomization	Infected sandflies were randomly selected to bite mice. All sandflies and their bites were analysed.
Blinding	No prior knowledge of the sandfly infection status was revealed to the researcher performing the RTqPCR.

Reporting for specific materials, systems and methods

We require information from authors about some types of materials, experimental systems and methods used in many studies. Here, indicate whether each material, system or method listed is relevant to your study. If you are not sure if a list item applies to your research, read the appropriate section before selecting a response.

Materials & experimental systems

n/a	Involved in the study	
☐	☒	Antibodies
☒	☐	Eukaryotic cell lines
☒	☐	Palaeontology
☐	☒	Animals and other organisms
☒	☐	Human research participants
☒	☐	Clinical data

Methods

n/a	Involved in the study	
☒	☐	ChIP-seq
☒	☐	Flow cytometry
☒	☐	MRI-based neuroimaging

Antibodies

Antibodies used	CCL3 (MIP-1a), CXCL2 (MIP-2) and IL-1b as part of a custom Bio-Plex Pro Mouse Cytokine Luminex assay (BIO-RAD UK)
Validation	http://www.bio-rad.com/webroot/web/pdf/lsr/literature/Bulletin_3156.pdf<br>http://www.bio-rad.com/webroot/web/pdf/lsr/literature/Bulletin_5297.pdf

Animals and other organisms

Policy information about studies involving animals; ARRIVE guidelines recommended for reporting animal research

Laboratory animals	Mouse, BALB/c, Female, Age 6-8 weeks, average weight 21g, supplier: Charles River UK.
Wild animals	The study did not involve wild animals.
Field-collected samples	The study did not involve samples from the field.
Ethics oversight	All animal experiments were carried out in accordance with the UK Animal Scientific Procedure Act (ASPA) 1986, which transposes European Directive 2010/63/EU into UK national law.<br><br>The animal studies were approved by the UK home office in granting Project licence 70/8427 under the Animal Scientific Procedure Act and all protocols had undergone appropriate local ethical review procedures by the Animal welfare and Ethical

Review Board (AWERB) of The London School of Hygiene and Tropical Medicine.

Note that full information on the approval of the study protocol must also be provided in the manuscript.
